# A multi-model genotype × environment interaction analysis discerning phenotypic plasticity of the strong culm trait in rice

**DOI:** 10.3389/fpls.2026.1727579

**Published:** 2026-05-13

**Authors:** Akshay Mamidi, Krishna Lavuri, Jyothi Badri, S. V. Sai Prasad, Raman Menakshi Sundaram

**Affiliations:** 1 Crop Improvement Section, Indian Council of Agricultural Research-Indian Institute of Rice Research (ICAR-IIRR), Hyderabad, India; 2 Department of Genetics and Plant breeding, Professor Jayashankar Telangana Agricultural University (PJTAU), Hyderabad, India

**Keywords:** culm strength, climate resilient rice, BLUPs, AMMI, GGE, lodging resistance, MTSI, stability analysis

## Abstract

A strong culm is a key trait for reducing lodging in rice, arising from the intricate coaction of morphological and environmental factors. As both grain yield (GY) and culm strength (CS) are complex traits, modeling genotype × environment interaction (G × E) and quantifying genotypic stability are essential for recommending genotypes in multi-environment trials (METs). We evaluated a selected set of recombinant inbred lines (RILs) derived from *indica* cv. Swarna and tropical *japonica acc.* IRGC 39111, along with checks across transplanted and direct-seeded environments, employing a multi-model approach to dissect mean performance and stability (MPS) for CS and GY traits. Phenotypic plasticity and stability were assessed using parametric and multivariate approaches, emphasizing AMMI, GGE, BLUPs, and the multi-trait stability index (MTSI). We further computed the weighted average of absolute scores (WAASB) to summarize G × E effects and WAASBY to integrate MPS. Selection was guided by genotype–ideotype distance via MTSI, and a genotype selection index (GSI) was used to synthesize yield and stability rankings. Breaking resistance (BR) was used as the primary proxy for CS. Significant G × E effects were detected for all traits. WAASB and WAASBY effectively captured stability and MPS, enabling the discrimination of broadly adapted and high-performing RILs. Multi-trait selection via MTSI improved selection efficiency relative to single-trait criteria and was consistent with AMMI-, GGE-, and BLUP-based inferences. The GSI identified RIL 315 (G16) as superior for GY and RIL 421 (G3) for BR. Consistently across AMMI, GGE, and MTSI, RIL 417 (G1), RIL 419 (G2), RIL 314 (G15), and tropical *japonica* acc. IRGC 10658 exhibited stable and high GY coupled with a strong culm across environments. A multi-model, multi-trait strategy robustly identified strong-culm, high-yielding RILs across variable environments. The convergence of WAASB/WAASBY, MTSI, and GSI supports the confident advancement of candidate lines and broadens the phenotyping framework and germplasm base for lodging resistance in future breeding programs.

## 1 Introduction

Lodging is a complex trait in rice influenced by several culm morphological, anatomical, and biochemical characteristics. The continuous translocation of organic matter from the culm sheath to the spikelets reduces the mechanical strength of the culm, while increased grain size and weight in the upper portions of the plant further elevate the risk of lodging (Ishimaru et al., 2008; Badri et al., 2024). A robust and plump culm provides better mechanical strength to the plant, allowing it to withstand environmental stress and maximizing its potential to bear heavy panicles. A holistic approach to breeding climate-resilient, high-yielding rice should place equal emphasis on strengthening culms while increasing yield, thereby preempting lodging.

The development of strong-culm cultivars is a major thrust area in rice research worldwide, as it represents an economical and effective strategy for managing lodging. A major hurdle in this endeavor is the complexity of the lodging phenomenon and the variable responses of the rice genotypes. Breeding rice cultivars with enhanced culm strength poses a major challenge due to the diverse and variable responses of rice genotypes. This entails validating trait stability across different environments or growing seasons characterized by varying weather conditions. The plasticity of plant responses for culm strength and yield stems from G × E interaction, which reduces the correspondence between phenotypic and genotypic values and introduces bias in estimating gene effects and combining ability for these traits. The need to model G × E interactions has led to the development of several mathematical models for deciphering G × E in multi-environment trials (METs). Based on inherent differences, these models are broadly classified into parametric and nonparametric approaches.

Parametric stability statistics in the univariate framework depend on distributional assumptions related to environmental, genotypic, and interaction effects. In contrast, the multivariate model aligns with the dynamic or agronomic concept of stability, employing parameters such as the regression coefficient (b_i_) (Finlay and Wilkinson, 1963), Shukla’s stability variance (*σ_i_^2^*) (Shukla, 1972), the regression coefficient (*b_i_*), and deviation from regression (*S_di_^2^*) (Eberhart and Russell, 1966; Perkins and Jinks, 1968). More recently, graphical methods such as the Additive Main Effects and Multiplicative Interaction Model (AMMI) and the Genotype + Genotype × Environment Interaction (GGE) model have become widely used. These approaches effectively visualize interaction patterns, identify stable genotypes across environments, and elucidate the relationships among genotype (G), environment (E), and G × E. AMMI, in particular, is effective because it partitions main and interaction effects, captures a large proportion of G × E variance, and facilitates genotype stratification through biplots (Gauch and Zobel, 1989, 1997; Crossa et al., 1990). Unlike ANOVA, which only identifies G × E as a significant source of variation, AMMI integrates an additive model (ANOVA) with principal component analysis (PCA), a multiplicative model to provide a more comprehensive depiction of G × E and its contributing environments. The AMMI stability value (ASV), derived from interaction principal component axes (IPCA1 and IPCA2), offers a reliable measure of genotypic stability and is comparable with the methods used by Shukla, Eberhart, and Russell for genotype stability (Purchase et al., 2000).

The GGE biplot (Yan and Tinker, 2006) complements AMMI by ranking genotypes within specific environments, identifying superior performers across mega-environments, and simultaneously assessing genotypic stability and mean performance. Furthermore, the need for multi-trait stability analysis led to the development of the multi-trait stability index (MTSI) (Olivoto et al., 2019). MTSI integrates factor analysis with genotype–ideotype distance. Since the genotype with the lowest MTSI is closest to the ideal, it performs better on average and is more stable across all of examined factors (Panda et al., 2023).

In parallel, nonparametric stability statistics, which require no primary assumptions, provide a useful alternative when breeders are primarily interested in rank-order differences across environments. They are robust to the addition or deletion of genotypes and are effective in capturing G × E patterns (Pour-Aboughadareh et al., 2022). Understanding the dynamic interplay between genotypes and environments (G × E interactions) is therefore central to breeding programs focused on developing stable, high-yielding lines. The tropical *japonica* subspecies represents an untapped reservoir of alleles for agronomically important traits such as strong culm. By merging genetic diversity across subspecies, breeders can generate enhanced variability, heterosis, and source–sink efficiency, which form the basis of the new plant type concept (Jyothi et al., 2018; Bagudam et al., 2021). In this study, a selected subset of RILs derived from the inter-subspecific cross Swarna (*indica*)/IRGC 39111 (tropical *japonica*) (INGR 23069) (Mamidi et al., 2024) was evaluated for stability across environments. These lines carry key QTLs for CS and GY, including *qCT7* for culm thickness, *qIBW7* for breaking resistance, *qSM8* for section modulus, *qCD3* for culm diameter, and *qSPY5* for grain yield (Mamidi et al., 2025). To dissect G × E and assess environmental responsiveness, robust multivariate techniques, including AMMI and the GGE biplot, were employed and complemented by WAASB and WAASBY indices to quantify stability. The present study aimed to identify genotypes with superior mean performance and stable expression of culm strength and yield traits across diverse environmental conditions. Additionally, MTSI, based on Euclidean distance, enabled simultaneous multi-trait selection. These objectives are grounded in the need to develop climate-resilient, high-yielding rice cultivars with enhanced lodging tolerance, addressing the complexity of lodging as a multifaceted trait influenced by both genetic and environmental factors. The convergence of findings across methods validated the identification of promising RILs with superior mean performance for CS and GY and consistent phenotypic expression across diverse environments. This integrated analytical framework not only enhances a nuanced understanding of G × E but also aids in identifying the environmental drivers of variation in culm strength, thereby facilitating the precise selection of genotypes tailored to specific growing environments for lodging resistance in rice.

## 2 Materials and methods

### 2.1 Experimental material

**Figure 1:**
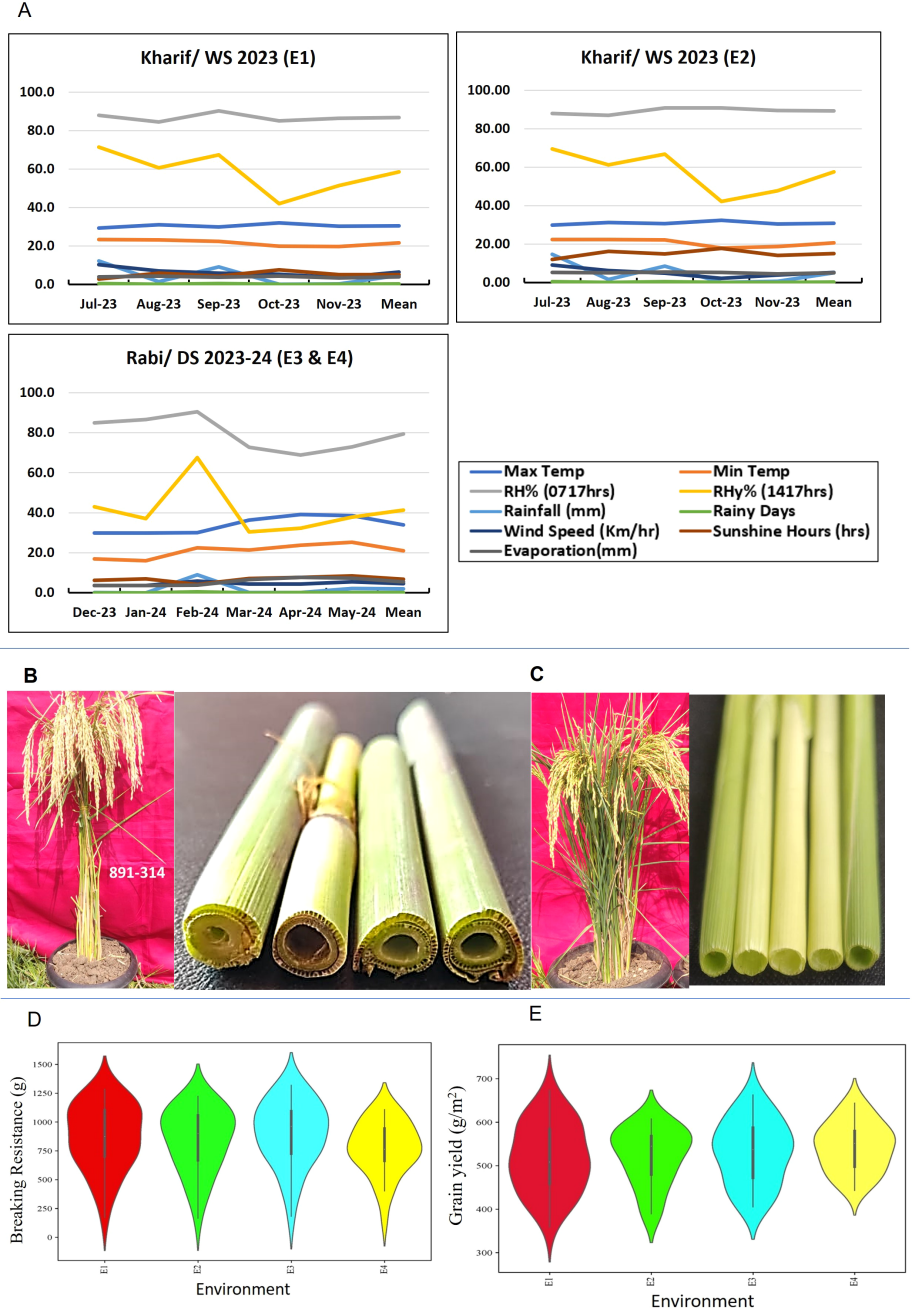
Environmental variation and phenotypic performance of rice genotypes across different growing seasons. **(A)** Monthly average weather parameters recorded during the rice crop growing periods across four environments: *Kharif* Wet Season 2023 (E1 and E2) and *Rabi* Dry Season 2023–24 (E3 and E4), **(B)** Field view of RIL-314 (Swarna/IRGC 39111) along with its culm cross sectional view, **(C)** Field view of weak culm parent-elite *indica* mega variety Swarna along with its culm cross sectional view, **(D, E)** Violin plots showing the distribution of breaking resistance (g) and grain yield (g/m²) of rice genotypes across environments. The width of each violin represents the density of data points, with broader sections indicating higher data concentration and tapering ends indicating lower density.

Studies were conducted at ICAR-IIRR to develop a mapping population of 353 RILs from the inter-subspecific cross between *Oryza sativa* cv. Swarna (*indica*) and IRGC 39111 (tropical *japonica* acc.). QTLs associated with CS and GY traits were identified based on multi-environment phenotyping and genotyping-by-sequencing of the RILs (Mamidi et al., 2025). In the present study, a subset of 20 RILs identified with superior performance for both CS and GY across environments was utilized for stability analysis. The experimental material comprised 30 genotypes (**Supplementary Table 1**), including 21 test genotypes and nine controls/checks. Test genotypes included 20 RILs derived from Swarna cv. *indica*/IRGC-39111 *acc.* Tropical *japonica* and one advanced breeding line (RNR 31479), while three popular high-yielding weak-culm cultivars (Swarna, Telangana Sona, Tellahamsa) served as lodging-sensitive (SC) checks and six strong-culm genotypes (IRGC39111, IRGC10658, RMS2085 (Kousik et al., 2024), RNR28361, JGL24423, and RNR29325) served as lodging-tolerant checks (TC). Stability for culm strength (CS) and grain yield (GY) were evaluated across environments under both transplanted (TP) and direct seeded rice (DSR) conditions (**Figure 1A**). Under TP conditions, field trials were conducted at the ICAR-Indian Institute of Rice Research (ICAR-IIRR) farm, Rajendranagar (latitude: 17°32′ N; longitude: 78°39′E), during *kharif* (wet season-WS) 2023 (E1); at the ICAR-IIRR farm located in International Crops Research Institute for the Semi-Arid Tropics (ICRISAT) campus, Patancheru (latitude: 17°53′N; longitude: 78°27′E), Hyderabad, Telangana, India, during WS 2023 (E2); and at the Institute of Rice Research (IRR) farm, Professor Jayashankar Telangana Agricultural University (PJTAU), Rajendranagar in *rabi* (dry season-DS) 2024 (E3). DSR experiments were conducted at the ICAR-IIRR farm, Rajendranagar, during DS2024 (E4). For TP environments, nursery beds were prepared following the recommended package of practices, and seeds of each genotype were sown in rows of 30 cm length. Nursery beds were irrigated at regular intervals to maintain sufficient moisture, and rice seedlings at 25–30 days after sowing were transplanted in two rows of 2 m length with a spacing of 15 cm × 10 cm in a randomized complete block design (RBD) with three replications. The recommended package of practices and fertilizer applications were followed to avoid yield loss. Direct-seeded rice was established using line sowing (20 cm row spacing), and fertilizers were applied at the recommended doses of N:P:K (150:50:50 kg/ha). A thin water layer was maintained at sowing, followed by alternate irrigation and drainage for seedling establishment and subsequent irrigation to a depth of 5 cm as required.

### 2.2 Data collection

#### 2.2.1 Measurement of yield parameters

The genotypes were evaluated for nine yield traits, namely: days to flowering (DFF), defined as the time taken for at least 50% of plants to flower from the date of sowing; plant height (PH) in cm, defined as the length between the plant base and the panicle tip; number of tillers (TN), defined as the total number of tillers per plant; number of productive tillers or panicle number (PN), defined as the number of panicle-bearing tillers; panicle length (PL) in cm, defined as the length of the panicle from base to tip; panicle weight (PW) in g, defined as the weight of a fully matured panicle; number of grains per panicle (GN), defined as the total number of filled grains in panicle; test weight (TW) in g, defined as the weight of 1,000 well-filled grains; and grain yield (GY) in g/m^2^, defined as the yield of all plants per m^2^ area. The traits PH, TN, PN, and PL were measured at maturity, whereas PW, GN, TW, and GY were recorded as postharvest observations.

#### 2.2.2 Measurement of culm morphology traits

Five primary culms per genotype were sampled at 20 days after heading (DAH) to assess culm morphological traits. Section modulus (SM), reflecting culm wall thickness and diameter, was estimated following the procedures described by Ookawa et al. (2010) and Badri et al. (2024). Culm diameter (CD) and culm thickness (CT) were measured as averages of the outer and inner diameters along the major and minor axes using digital Vernier calipers.

Culm strength (CS) traits were evaluated using a prostrate tester (DIK-7400, Daiki Rika Kogyo Co. Ltd., Tokyo, Japan) to determine bending stress (BS) as described by Hai et al. (2005), and the bending moment at breaking (M) was calculated following the method of Jyothi et al. (2018). Basal internode length (IL) was recorded as the length of the second lower internode from the soil surface. Internode breaking weight (IBW), an indicator of culm breaking resistance (BR), was measured according to Badri et al. (2024) and Mamidi et al. (2025) using a horizontally mounted loading apparatus with incremental loading until the internode collapsed.


$$\text{Section modulus }(\text{SM})=\;\frac{\text{π}}{32}\;\times ({\text{a}}_{1}^{3}{\text{b}}_{1}-{\text{a}}_{2}^{3}{\text{b}}_{2})$$

where *a_1_* is the outer diameter of the minor axis in the oval cross-section, *b_1_* is the outer diameter of the major axis of the oval cross-section, *a_2_* is the inner diameter of the minor axis of the oval cross-section, and *b_2_* is the inner diameter of the major axis in the oval cross-section.


$$\text{Bending stress }(\text{BS})=\;\frac{\text{TR}}{40}\;\times \frac{1000}{\text{TN}}$$

where TR is the prostrate tester reading value (measure of pushing resistance-PR) and TN is the tiller number.


Bending moment at breaking(M)=SM×BS

### 2.3 Stability analysis

Descriptive statistics were computed for all traits to summarize phenotypic variation across genotypes and environments. The Shapiro–Wilk test was performed to assess the normality of the data across test environments, and Bartlett’s test was used to assess homoscedasticity. Phenotypic data were analyzed using both model-based and classical statistical approaches. Model-based analyses were conducted using a linear mixed model implemented under restricted maximum likelihood (REML) estimation through the “gamem” (Genotype × Environment Analysis using Mixed Effects Model) procedure, treating genotypes, environments, and genotype × environment (G × E) interactions as random effects, while replication was included as a random factor nested within environments. This approach allowed appropriate partitioning of phenotypic variance while accounting for experimental replication and environmental heterogeneity.

Variance components obtained from the mixed model were used to estimate broad-sense heritability (H²), H2 = σ_g_^2^/[σ_g_^2^ + (σ_g_ × e^2^/e) + (σ_e_^2^/re)], accounting for genotypic variance, G × E interaction variance, residual variance, number of environments, and number of replications. Best Linear Unbiased Predictions (BLUPs) of genotypic effects were extracted from the mixed model and used to rank genotypes for overall performance across environments.

Furthermore, Additive Main Effects and Multiplicative Interaction (AMMI) and Genotype plus Genotype × Environment (GGE) biplot analyses were performed using environment-centered genotype means assuming genotypes as fixed effects. These analyses were used for graphical and descriptive exploration of genotype × environment interaction patterns.

For model-based assessment of stability, the Weighted Average of Absolute Scores from BLUP-based AMMI (WAASB) was computed using REML-derived G × E BLUPs. The Multi-Trait Stability Index (MTSI) was employed to enable simultaneous selection of genotypes with desirable performance and stability across multiple traits by integrating WAASB values and trait-specific desirability criteria. In addition to the model-based analysis, various parametric statistics were employed to assess genotype performance across diverse environments. The Annicchiarico environmental index (AEI) (Carneiro et al., 2019), which incorporates mean performance and measures of variation to assess how a genotype responds to environmental changes in terms of stability and adaptability, was used to predict favorable or unfavorable environments and identify the most suitable genotype for a particular environment. The contribution of each genotype to the G × E sum of squares was assessed in terms of ecovalence (W^2^) (Wricke, 1962). The W^2^ of the *i*th genotype represents its interaction with the environments, squared and summed across environments. Genotypes with lower values, indicating smaller deviations from the mean across environments, were identified as stable genotypes.


$${\text{W}}^{2}=\sum ({\text{X}}_{\text{ij}}-\bar{{\text{X}}_{\text{i}.}}-\bar{{\text{X}}_{.\text{j}}}+{\bar{{\text{X}}_{.})}}^{2}$$

where *X_ij_* is the mean trait value of the *i*th genotype in the *j*th environment; *X_i_* is the mean trait value of the *i*th genotype; *X_j_* is the mean trait value of the *j*th environment; and *X.* is the grand mean. Shukla’s stability variance (σ^2^) (Shukla, 1972) for the *i*th genotype was calculated as its variance across environments after removing the main effects of environmental means. Genotypes with lower values were considered more stable.


$${\text{σ}}^{2}=\lfloor \frac{\text{p}}{\left(\text{p}-2)(\text{q}-1\right)}\rfloor {\text{W}}^{2}-\;\frac{\sum {\text{W}}_{\text{i}}^{2}}{\left(\text{p}-1)(\text{p}-2)(\text{q}-1\right)}$$

where *W^2^* is Wricke’s ecovalence, and *p* and *q* are the numbers of genotypes and environments, respectively. The P_i_ (Performance Index) (Kamidi, 2001), a composite index that combines multiple traits, including yield, across different environments and reflects the average performance of a genotype in both favorable and unfavorable environments was calculated as


$$P_{i_{a}}=\;\frac{1}{n}\sum Y_{i}$$

where $\;Y_{i\;}$ is the yield of the genotype in the *i*th environment; n is the number of environments. This was used to measure the sensitivity of a genotype to changes in the environment. A higher value indicates greater sensitivity to environmental changes, while a lower value indicates greater stability. Pinthus’s coefficient of determination (R^2^) (Pinthus, 1973), the variation in mean yield explained by genotype response across environments, was measured as


$$R^{2}=\frac{b_{i}^{2}\sum_{k\;=\;0}^{n}(\bar{{\text{X}}_{.\text{j}}}-{\bar{{\text{X}}_{.})}}^{2}}{\sum_{k\;=\;0}^{n}({\text{X}}_{\text{ij}}-{\bar{{\text{X}}_{\text{i}.})}}^{2}}$$

where *b_i_* is regression slope, $X_{ij}$ is the grain yield of the *i*th genotype in the *j*th environment; $\bar{X_{i.}}$ is the mean grain yield of the *i*th genotype; $\bar{X_{.j}}$ is the mean grain yield of the jth environment; and $\bar{X_{.}}$ is the grand mean. Genotype with highest value was considered as stable genotype.

Multi-model stability analyses were performed using GGE biplots and AMMI in R Studio *v*.4.3.2. The metan package facilitated GGE biplots and AMMI analysis, generated biplot visualizations, computed BLUPs, WAASB, WAASBY, and MTSIs, and calculated the percentage of G × E interaction from the total sum of squares. GGE biplots and AMMI graphically showcased G × E interaction and genotype ranking based on mean performance and stability. AMMI biplots were constructed based on genotype mean performance versus the first principal component axis (PCA1) and between the first two principal component axes (PCA1 vs. PCA2)


$$Y_{ij}=\mu +\delta_{i}+\beta_{j}+\sum_{k\;=\;1}^{k}\lambda_{k}\delta_{ik}\beta_{jk}+\epsilon_{ij}$$

where *Y_ij_* is the mean yield of the *i*th genotype (*I* = 1, 2,…., k) in the *j*th environment (*j* = 1,2,….,k); *μ* is the general mean; $\delta_{i}$ is the *i*th genotype effect; $\beta_{j}$ is the *j*th location effect; $\lambda_{k}$ is the eigenvalue of the *k*th P CA axis; $\text{and }\epsilon_{ij}$ is the residual. The extent of interaction was reflected by the distance of the environment and genotype vectors from the biplots origin. Longer vectors suggested interaction, while shorter vectors suggested less interaction, serving as a reliable indicator for selecting genotypes with consistent performance and adaptability. Further, genotypes were ranked based on AMMI stability statistics. The average squared eigenvector value (EV) parameter proposed by Zobel (1994) was calculated as


$${\text{EV}}_{\text{i}}=\sum_{n=1}^{N}\;\frac{\gamma_{in}^{2}}{N}$$

For EV_1_, N is one; for EV_V_, N is the number of IPCs retained in the AMMI model *via* validation procedures; and for EV_F_, N is the number of IPCs retained in the AMMI model *via* F-test. The genotypes with the lowest values for these statistics were identified as the most stable. The Sums of the Absolute Value of the IPC Scores (SIPC) (Sneller et al., 1997) were calculated as the sum of the absolute values of the IPC scores for each test genotype as


$$\text{SIPC}=\sum_{n=1}^{N}\left\lfloor \gamma_{n}^{0.5}\gamma_{in}\right\rfloor$$

Similar to the EV statistic, for SIPC_1_, N is one; for SIPC_v_, N is the number of IPCs that are retained in the AMMI model *via* validation procedures; for SIPC_F_, N is the number of IPCs retained in the AMMI model *via* F-tests. *λ_n_* and *γ_in_* are the eigenvalue of the *n*th IPCA that is retained in the AMMI model and the eigenvector for the *i*th genotype from the *n*th IPCA, respectively. The lowest values for these statistics indicated the highest stability. Furthermore, AMMI-based stability statistics represent the sum across environments (AMGE) of the G × E interaction modeled by AMMI.


$$\text{AMGE}=\sum_{N}^{M}\sum_{n=1}^{M}\left[\lambda_{n}\gamma_{in}\delta_{jn}\right]$$

where *λ_n_, γ_in_*, and *δ_jn_*are the eigenvalue of the *n*th interaction PCA (IPCA) retained in the AMMI model, the eigenvector for the *i*th genotype from the *n*th IPCA, and the eigenvector for the *j*th environment from the *n*th IPCA, respectively. For AMGE_1_, N is one; for AMGE_V_, N is the number of IPCs retained in the AMMI model *via* validation procedures; for AMGE_F_, N is the number of IPCs retained in the AMMI model *via* F-tests. Lower values of these statistics indicated highest stability.

AMMI-based statistics using the distance of the IPCA point from the origin in space (D) (Annichiarico, 1997) provided the G×E estimate of a particular genotype with a group of environment samples. The greater the D value of a genotype, the greater the distance of the genotype from the IPCA origin. Thus, the genotype with the lowest value of this statistic would be the most stable.

${\text{D}}_{\text{a}}={\left\lfloor \sum_{n=1}^{N}{\left(\lambda_{n}\gamma_{in}\right)}^{2}\right\rfloor }^{0.5}$


$${\text{D}}_{\text{z}}={\left[\sum_{n=1}^{N}{\left(\gamma_{in}\right)}^{2}\right]}^{0.5}$$

where *λ_n_* and *γ_in_*are the eigenvalue of the *n*th IPCA retained in the AMMI model and the eigenvector for the *i*th genotype from the *n*th IPCA, respectively.

The AMMI stability value (ASV) is the distance from a coordinate point to the origin in a two-dimensional scatterplot of IPCA1 scores against IPCA2 scores (Purchase et al., 2000)


$$\text{ASV}=\sqrt{[\frac{\text{PCA}1_{\text{ss}}}{\text{PCA}2_{\text{ss}}}+(\text{PCA}1_{\text{score}})]+{\left(\text{PCA}2_{\text{score}}\right)}^{2}}$$

where $\text{PCA}1_{\text{ss}}$/ $\text{PCA}2_{\text{ss}}$ is the ratio between the sum of squares of the 1st and 2nd interaction PCs, and IPCA1 and IPCA2 are the genotypic scores of these components in the AMMI model. The genotype with the lowest value of this statistic is considered more stable. Conversely, a larger absolute PCA indicates greater adaptability of a specific genotype to a specific environment.

Wricke’s ecovalence (W^2^), based on the fitted AMMI model [W(AMMI)] (Raju, 2002), was used as another stability measure,


$${\text{W}}_{\left(\text{AMMI}\right)}=\sum_{n=1}^{N}\left(\lambda_{n}^{2}\gamma_{in}^{2}\right)$$

*λ_m_* and *γ_in_* are the singular value for the PCA axis and the IPCA score of the *i*th genotype for that axis, respectively. N is the number of significant IPCAs. Therefore, the stability rank order obtained from W_(AMMI)_ is equivalent to that of W^2^. The genotype with the lowest value of statistic is the most stable. The AMMI-based stability parameter (ASTAB) (Rao and Prabhakaran, 2005) was calculated as


$$\text{ASTAB}=\sum_{n=1}^{N}\left\lfloor \lambda_{n}\gamma_{in}^{2}\right\rfloor$$

where *λ_n_* and *γ_in_* are the eigenvalue of the *n*th IPCA and the eigenvector value of the *i*th genotype, respectively. A genotype is considered more stable when the value of this statistic is low.

The modified AMMI stability values (MASV) (Zali et al., 2012) were estimated as


$$\text{MASV}=\sqrt{\sum_{n=1}^{N-2}\;{\left((\frac{{\text{SSIPCA}}_{\text{n}}}{{\text{SSIPCA}}_{\text{n }+\;1}})({\text{IPCA}}_{\text{n}})\right)}^{2}+{\left({\text{IPCA}}_{\text{N}}\right)}^{2}}$$

Similar to ASV, the genotype with the lowest value of MASV value, which uses all significant IPCAs in the MASV statistic, was selected as the most stable. The absolute value of the relative contribution of IPCA (Z_a_) and the absolute value of genotype × environment interaction modeled by AMMI [AV_(AMGE)_] were measured as described by Zali et al. (2012).


$${\text{Z}}_{a}=\sum_{i=1}^{n}\left\lfloor \theta_{n}\gamma_{in}\right\rfloor$$

where *θ_n_* is the percentage of the sum of squares explained by the *n*th IPCA, and N is the number of IPCs retained in the AMMI model *via* the F-test. Higher stability is indicated by lower Z_a_ values.


$${\text{AV}}_{\left(\text{AMGE}\right)}=\sum_{j=1}^{E}\sum_{n=1}^{N}\left\lfloor \lambda_{n}\gamma_{in}\delta_{jn}\right\rfloor$$

where N is the number of significant IPCs retained in the AMMI model via the F-test and *λ_n_, γ_in_*, and *δ_jn_* are the eigenvalue of the *n*th interaction PCA (IPCA) retained in the AMMI model, the eigenvector of the *i*th genotype from the *n*th IPCA, and the eigenvector of the *j*th environment from the *n*th IPCA, respectively. The most stable genotype is the one with the lowest AV_(AMGE)_ value. The AMMI stability index (ASI) (Jambhulkar et al., 2014) was estimated as


$$\text{ASI}=\sqrt{{\left(\text{IPCA}1\times {\text{θ}}_{1}^{2}\right)}^{2}+{\left(\text{IPCA}2_{\text{score}}\times {\text{θ}}_{2}^{2}\right)}^{2}}$$

where *θ_2_^1^* and *θ_2_^2^* are the percentages of the sum of squares explained by the first two IPCA effects, respectively, and IPCA1 and IPCA2 are the scores of the first two PC interactions. The lowest value of this statistic was considered the most stable.

The modified AMMI stability index (MASI) (Ajay et al., 2018) was computed considering all significant IPCAs in the AMMI model.


$$\text{MASI}=\sqrt{\sum_{\text{n}=1}^{\text{N}}{\left(({\text{IPCA}}_{\text{n}})\right)}^{2}\times }\theta_{\text{n}}^{2}$$

IPCA_n_ and $\theta_{n}^{2}\;$are the scores of the *n*th IPCA and the percentage of the sum of squares explained by the *n*th IPCAs effects, respectively. Similarly, lower values of this statistic show the most stability.

The Genotype Stability Index (GSI) (Farshadfar, 2008), a nonparametric statistic, was based on the rank of the mean yield of a genotype across environments (*r*Y) and the rank of the ASV value (*r*ASV). GSI was calculated as GSI = rASV + rY, and the genotype with the lowest GSI value was considered as more stable.

GGE biplots were constructed according to the model (Yan et al., 2000; Yan, 2002; Yan and Kang, 2002; Yan and Tinker, 2006) based on the singular value decomposition (SVD) of the first two principal components, ignoring random error. The site regression model, a multiplicative model in the bilinear terms, shows the main effects of genotype plus the genotype × environment interaction (GGE).


$$Y_{ij\;-\;}\mu_{j}=\sum_{k\;=\;1}^{k}\lambda_{k}\delta_{ik}\beta_{jk}+\epsilon_{ij}$$

The biplots were constructed using two axes: PC1 denotes the magnitude of the trait under study, and PC2 measures stability. The ranking of genotypes was allocated in increasing order of each stability parameter. The biplots were based on singular-value partitioning (partitioning = 2), transformed (transform = 0), environment-centered (centering = 2), and standard deviation-standardized (scaling = 0).

Best linear unbiased prediction (BLUP) is considered the best method for estimating random effects in a linear model (Smith et al., 2005). The three parameters—HMGV (harmonic mean of genotype values), RPGV (relative performance of genotypic values), and HMRPGV, which considers stability, adaptability, and mean performance simultaneously—were calculated as


$$\text{HMGV}\;=\frac{E}{\sum_{j\;=\;1}^{E}\frac{1}{GV_{ij}}};\text{ RPGV}=\frac{1}{E}\sum_{j\;=\;1}^{E}\frac{GV_{ij}}{\mu_{j}};\text{ HMRPGV}=\frac{E}{\sum_{j\;=\;1}^{E}\frac{1}{GV_{ij}/\mu_{j}}}$$

where $GV_{ij}$ are the genotypic values (BLUP) for the ith genotype in the jth environment; $\mu_{j}$ is the mean of environment j; and E is the number of environments. Higher values of these parameters are desirable. Olivoto et al. (2019) combined features of both AMMI and BLUP models and developed a unique stability parameter named WAASB. The stability of each genotype was quantified by the weighted average of absolute scores (WAAS) from the singular value decomposition of the matrix of best linear unbiased predictions for the G × E effects generated by a linear mixed-effect model (WAASB). Simultaneous selection for mean performance and stability was performed using the WAASBY index, which weights mean performance and stability (WAASB). This statistic is the weighted average of absolute scores from the singular value decomposition of the matrix of BLUPs for the G × E interaction effects generated by a linear mixed-effect model.


$$\text{WAASB}=\frac{\sum_{k=1}^{p}\lfloor {\text{IPCA}}_{\text{ik}}\times {\text{EP}}_{\text{k}}\rfloor }{\sum_{k=1}^{p}\lfloor {\text{EP}}_{\text{k}}\rfloor }$$

where IPCA*_ik_* is the score of the *i*th genotype (or environment) in the *k*th IPCA, and EP_k_ is the proportion of variance explained by the *k*th IPCA. The genotype with the lowest WAASB value is considered the most stable. Furthermore, to identify superior performers and stable genotypes, a biplot based on WAASB and the trait was used. In the Multi-trait Stability Index (MTSI), the stability of each genotype was measured by estimating the weighted average of absolute scores from the singular value decomposition of the matrix of BLUPs for the GEI effects generated by a linear mixed-effects model (LMM), and simultaneous selection for mean performance and stability was performed using the WAASBY index, weighing mean performance (Y) and stability (WAASB).


$${\text{MTSI}}_{\text{i}}=\sum_{\text{j}=1}^{\text{f}}{\left[{\left({\text{F}}_{\text{ij}}-{\text{F}}_{\text{j}}\right)}^{2}\right]}^{0.5}$$

where MTSI is the multi-trait stability index for the *i*th genotype, *F_ij_* is the *j*th score of the *i*th genotype, and *F_j_* is the *j*th score of the ideotype. The genotype with the lowest MTSI value is closer to the ideotype and therefore exhibits high mean performance and stability across environments for all traits studied. Desirable genotypes with maximum productivity coupled with high stability were selected with a 15% selection intensity. These selected and nonselected genotypes were shown graphically by plotting MTSI scores. GSI was estimated as described by Farshadfar (2008). All statistical analyses were performed using R version 4.3.2, primarily employing the *metan* package, along with supporting packages for data visualization and output generation.

## 3 Results

### 3.1 Mean performance of the genotypes and implications of environmental factors

Grain ripening and maturity in WS 2023 at Rajendranagar (E1) and Ramachandrapuram (E2) under transplanted (TP) conditions coincided with rainfall and higher wind speed. The crop experienced bright sunshine hours from tillering to maturity in DS 2024 at IRR-PJTAU under TP conditions (E3) and DS 2024 at Rajendranagar under DSR conditions (E4), along with high wind speed. The mean evaporation rate was highest in E4 and E3 (**Figure 1A**). Among the test environments, E2 exhibited the longest crop growth period relative to the other three environments, attributable to delayed transplanting. The transplanting of overaged seedlings in E2 further contributed to a comparatively lower mean yield in this environment.

A significant contrast between TP and DSR for culm strength (CS) and grain yield (GY) traits was observed, primarily attributable to differences in establishment patterns and moisture regimes. The violin plots (**Figures 1B, C**) elucidated differences in central tendencies and distribution shapes, offering valuable insights into the impact of transplanted and DSR conditions on BR and GY. All traits exhibited a normal distribution, as verified by Shapiro–Wilk test, while Bartlett’s test indicated homogeneity of data within individual environments, although it revealed heteroscedasticity across environments. The CS component traits displayed mean values of basal internode length (IL)—11 cm; culm length (CL)—76.24 cm; culm diameter (CD)—6.13 mm; culm thickness (CT)—1.83 mm; section modulus (SM)—17.28 mm^3^; pushing resistance (PR)—21.26; bending stress (BS)—48.22 N/m^2^; bending moment at breaking (M)—825 gfcm; culm with leaf sheath weight (CLSW)—10.57 g; and breaking resistance (BR)—847 g. The mean values of GY-related traits were plant height (PH)—106 cm, tiller number (TN)—12.5, panicle number (PN)—10.17, panicle weight (PW)—4.14 g, thousand grain weight (TW)—20.32 g, grain number (GN)—218 and grain yield (GY)—527 g/m^2^. CV% was within 20% for all traits except for M, which reached 25.54%. These findings indicate substantial variability in the CS and GY-related traits among the genotypes. Across environments, BR was the highest in RIL 314 (G15) (1,065 g; **Figure 1D**), whereas the susceptible check, Swarna (G21), displayed the lowest BR (169 g; **Figure 1E**). RIL 315 (G16) also exhibited highest mean GY (635 g/m^2^), while RIL-214 (G10) and the breeding line RNR 31479 (G30) exhibited comparatively lower yields (431 g/m^2^). The overall mean BR across environments was 847 g, with the maximum recorded in E3 (900 g) and the lowest in E4 (775 g). Similarly, the mean GY across environments was 527 g/m^2^, with the highest in E1 (547 g/m^2^) and the lowest in E4 (513 g/m^2^) (**Table 1**).

**Table 1:** Descriptive statistics across environments and environment wise mean trait values across four environments.

Parameters	PH	IL	CL	TN	PR	CD	CT	SM	BS	M
Mean ± SE	105.76 ± 0.77	11.18 ± 0.18	76.24 ± 0.98	12.5 ± 0.2	21.26 ± 0.35	6.13 ± 0.06	1.83 ± 0.03	17.28 ± 0.43	48.22 ± 1.16	824.56 ± 9.35
CV%	12.59	13.94	14.41	10.52	12.05	7.60	12.69	18.24	15.80	25.54
Min ENV	E2 (103)	E2 (10.93)	E2 (74.74)	E2 (12.11)	E2 (21)	E4 (5.81)	E4 (1.66)	E4 (15.05)	E4 (44.82)	E4 (729.53)
Max ENV	E3 (108)	E3 (11.38)	E3 (78.54)	E3 (12.8)	E3 (22)	E3 (6.41)	E3 (1.94)	E3 (18.46)	E3 (50.85)	E3 (880.52)
MinGEN	G16 (89)	G15 (5.49)	G30 (45.42)	G25 (7.43)	G21 (12)	G21 (2.68)	G21 (0.59)	G21 (1.36)	G21 (13)	G21 (17)
Max GEN	G2 (126)	G20 (16.32)	G2 (103.8)	G21 (20.5)	G14 (33)	G1 (7.78)	G2 (2.82)	G2 (32.25)	G11 (82)	G14 (1735)
E1	106.3	11.3	76.7	12	21.4	6.25	1.89	18.08	49.33	859
E2	103.4	10.9	74.7	12	20.8	6.07	1.83	17.53	47.88	829
E3	108.9	11.4	78.5	13	22.0	6.41	1.94	18.46	50.85	881
E4	104.5	11.2	75.0	10	20.9	5.81	1.66	15.05	44.82	730
	BR (IBW)	CLSW	DFF	PN	PL	GN	PW	TW	GY	
Mean ± SE	847.05 ± 4.94	10.57 ± 0.18	122.25 ± 0.82	10.17 ± 0.17	21.78 ± 0.18	218.31 ± 2.48	4.14 ± 0.05	20.31 ± 0.28	527.66 ± 4.14	
CV%	13.48	13.68	3.92	13.41	10.31	11.62	17.49	16.61	9.07	
Min ENV	E4 (775.33)	E4 (10.22)	E4 (118.05)	E2 (9.91)	E4 (21.26)	E2 (212)	E2 (3.95)	E2 (19.32)	E4 (513.67)	
Max ENV	E3 (901.29)	E3 (10.99)	E3 (127.04)	E3 (10)	E3 (22.6)	E3 (223)	E4 (4.36)	E4 (21.3)	E1 (547.11)	
MinGEN	G21 (169)	G21 (4.91)	G30 (99.52)	G23 (6)	G5 (17.26)	G20 (143)	G25 (2.58)	G9 (21.74)	G30 (431)	
Max GEN	G15 (1065)	G23 (15)	G21 (147.7)	G21 (19)	G23 (28)	G1 (285)	G23 (6.57)	G4 (27.86)	G16 (635)	
E1	868	10.68	124	10	23.8	221	4.08	20.01	547	
E2	844	10.38	130	10	23.0	218	4.26	21.30	521	
E3	901	10.99	127	10	24.1	223	4.16	20.61	530	
E4	775	10.22	118	8	22.8	211	3.95	19.32	513	

CV, coefficient of variation; SE, standard error; ENV, environment; GEN, genotype; E1, E2, E3, E4, Environment 1, 2, 3, and 4, respectively. PH, plant height (cm); IL, internode length (cm); CL, culm length (cm); ODMa, outer diameter of major axis(mm); ODMi, outer diameter of minor axis(mm); IDMa, inner diameter of major axis (mm); IDMi, inner diameter of minor axis (mm); TN, tiller number; PR, pushing resistance; CD, culm diameter(mm); CT, culm thickness (mm); SM, section modulus (mm^3^); BS, bending stress (g/mm^2^); M, bending moment at breaking (g cm); BR, breaking resistance (g) as a measure of internode breaking weight (IBW); CLSW, culm with leaf sheath weight (g); DFF, days to 50% flowering; PN, panicle number; PL, panicle length (cm); GN, grain number; PW, panicle weight(g); TW, test weight(g); GY, grain yield (g/m^2^).

### 3.2 Association among strong culm, yield, and their component traits

**Figure 2:**
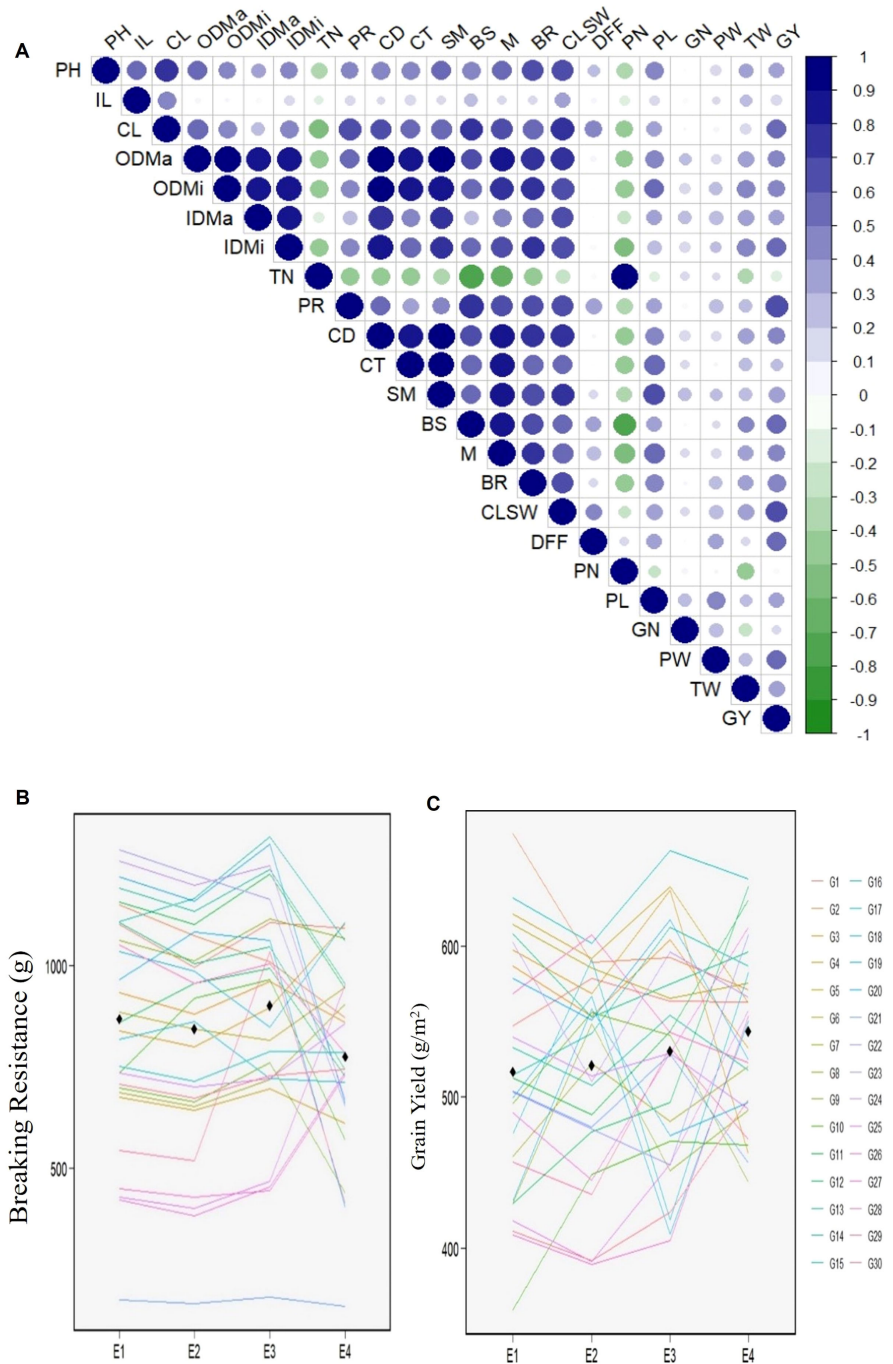
Trait correlations and cross over interactions of RILs (Swarna/IRGC 39111) and GY-CS checks across environments **(A)** Pearson correlation of pooled mean across four environments for mechanical traits related to culm strength and grain yield traits. Color (blue, positive correlation; green, negative correlation) intensity and circle size are proportional to the correlation coefficient, **(B, C)** Plots depicting rank-order interaction of genotype performance of breaking resistance and yield traits respectively at each environment under study.

Correlation analysis among the studied traits showed that BR had a strong, significant positive association with components of CS traits—CD (0.73***), CT (0.56**), SM (0.69***), BS (0.63***), M (0.70***), CLSW (0.65***), PR (0.64***), and PH (0.63***)—while the association with IL was not significant (**Figure 2A**). This highlights the strong association between culm morphological and physical strength traits. Furthermore, TN (−0.47*) was negatively correlated with culm morphological traits and BR. Additionally, a strong positive association was observed between BR and GY *per se* (0.48**). These findings indicate that CS traits exhibit a synergistic relationship with GY, whereas with PN and TN they show a trade-off. Having identified a strong association between BR and GY and recognizing their complex nature, these two traits were chosen to further investigate their G × E interaction using multimodel analysis, to elucidate variation in CS and GY among genotypes, and to identify HY–CS stable rice genotypes across varying environmental conditions.

### 3.3 Variance analysis for strong culm and grain yield

The combined ANOVA (**Table 2**) revealed highly significant differences (p ≤0.001) for both BR and GY across environments, genotypes, and their interactions. These differences can be attributed to dynamic environmental conditions, inherent genetic diversity, and their interactive effects. The partitioning of variance indicated that, for BR, 18.28% of the total sum of squares (TSS) was explained by the environment, 14.83% by genotype, and the largest portion, 44.70%, by the G × E interaction. These results demonstrate the significant role of G × E in shaping the genotypic response for BR. For GY, both genotype and interaction effects were highly significant (p <0.001). The relative contribution of the environment factor was 11.53%, the genotype factor was 21.24%, and G × E accounted for 30.08% of the TSS.

**Table 2:** Estimation of significant level for breaking resistance and grain yield across four environments revealed by ANOVA.

Trait	Source	df	Sum Sq	Mean Sq	F value	Pr(>F)	Proportion	Accumulated
Breaking resistance	ENV	3	7,680,347	2,560,116**	3.48	0.01	18.28	
	REP(ENV)	8	588,620	73,578***	6.86	4.3E−08	1.4	
	GEN	29	6,228,001	214,759***	60.43	1.023E−91	14.83	
	GEN: ENV	87	18,794,226	216,026***	6.67	3.832E−31	44.7	
	PC1	31	5,306,858	171,189***	15.96	0	85.2	85.2
	PC2	29	754,238	26,008***	2.42	0.0002	12.1	97.3
	PC3	27	166,904	6,182**	0.58	0.0437	2.7	100
	Residuals	232	2,488,239	10,725				
	Total	446	42,007,434	94,187				
Grain Yield	ENV	3	373,898	124,633**	0.28	0.02	11.53	
	REP(ENV)	8	355,922	44,490***	64.53	9.3E−55	1.09	
	GEN	29	688,777	23,751***	48.80	1.715E−82	21.24	
	GEN: ENV	87	975,696	11,215***	11.48	9.682E−50	30.08	
	PC1	31	392,552	12,663***	18.37	0.000	57	57
	PC2	29	205,988	7,103***	10.3	0.000	29.9	86.9
	PC3	27	90,237	3,342***	4.85	0.000	13.1	100
	Residuals	232	159,961	689				
	Total	446	3,243,029	7,271				

df, degrees of freedom; ** significant at *p ≤*0.05; *** significant at *p ≤*0.001; Sum Sq, sum of squares; Mean Sq, mean sum of squares; ENV, environment; GEN, genotype; REP, replication; GEN: ENV- G × E interaction; PC1, 2, 3 are the first three interaction principal components, respectively.

The presence of qualitative (crossover-type) interaction was evident, as the rank order of genotypes varied across environments (**Figures 2B, C**), further indicating a substantial G × E effect. To evaluate environmental favorability and genotype adaptability, the Annichiarico environmental index (AEI) was applied, allowing classification of environments as favorable or unfavorable and identification of the most responsive genotype (**Supplementary Table 3**). Based on AEI, E1 and E3 were identified as favorable environments for both BR and GY, whereas E2 and E4 represented more challenging conditions. These findings reinforce the presence of strong G × E interactions influencing genotypic performance across environments. PCA further provided deeper insights into G × E interaction patterns. For BR, ordination with an approximate F-statistic revealed significant distinctions across PC1 (87.9%) and PC2 (9.8%), totaling 97.7% of the variation. In the case of GY, PC1 (57.0%) and PC2 (29.9%) accounted for a cumulative variance of 86.9%, with both axes contributing significantly (P <0.001) (**Table 2**).

### 3.4 AMMI biplot analysis

#### 3.4.1 Additive main effects and multiplicative interaction (AMMI 1)

**Figure 3:**
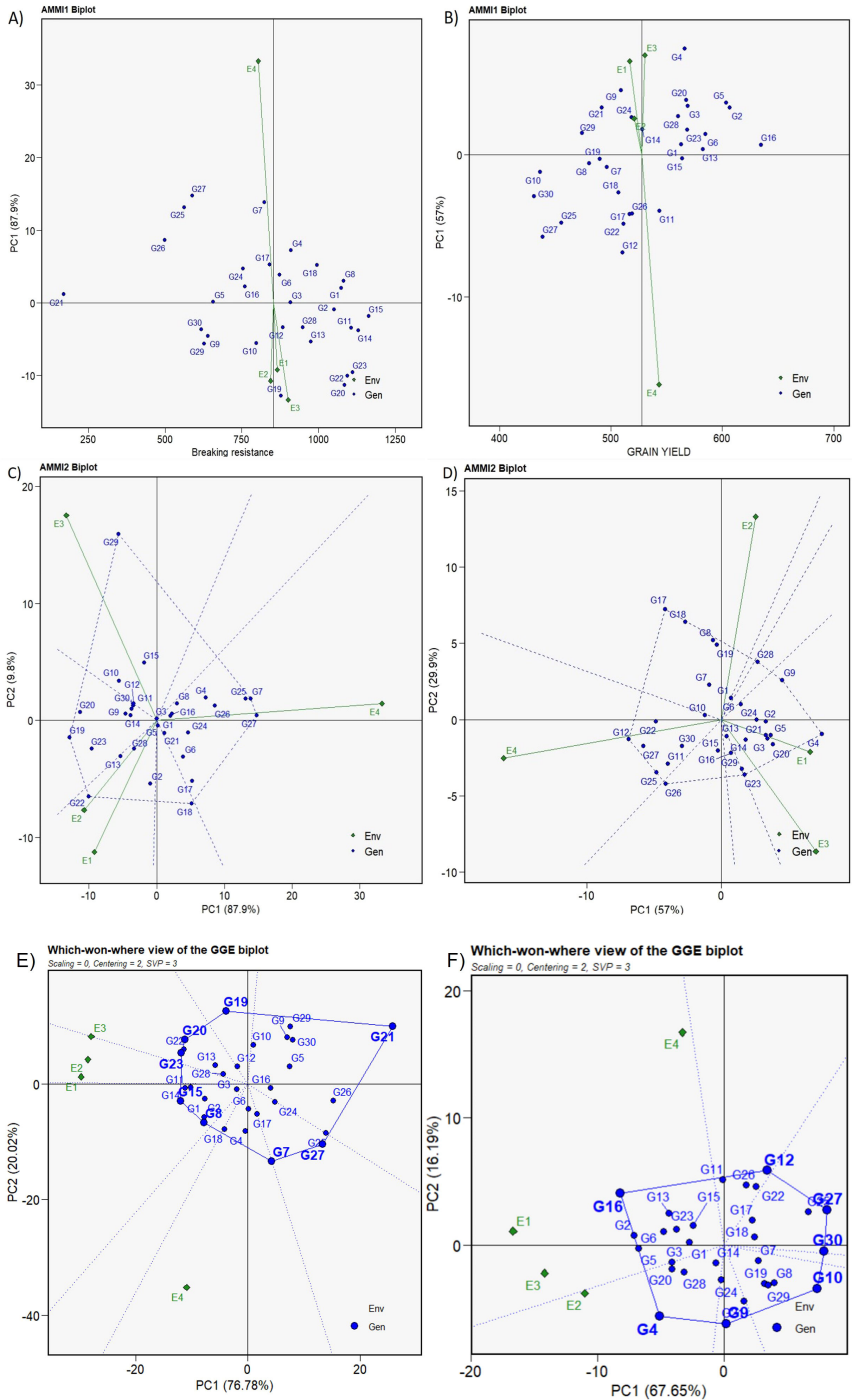
AMMI and Which-won-where biplots for breaking resistance and grain yield among 30 studied genotypes across four environments. **(A)** The “AMMI 1” biplot depicting the primary effects of BR and PC1 impacts of both genotype and environment, **(B)** The “AMMI 1” biplot depicting the primary effects of GY and PC1 impacts of both genotype and environment, **(C)** The “AMMI 2” biplot visualizing the combined effects of the PC1 and PC2 for genotypes along with the G × E interaction impact for BR, **(D)** The “AMMI 2” biplot visualizing the combined effects of the PC1 and PC2 for genotypes along with the G × E interaction impact for GY, **(E, F)** “Which-won-where” biplots illustrating the combined effects of genotype main effects and G × E interaction for BR and GY, respectively.

The biplot displays the PC1 term on the abscissa (differences in main effects) and trait interaction effects (differences in interaction patterns) on the ordinate. For both BR and GY, all environments were displayed as vectors parallel to PC1, indicating substantial interaction effects (**Figures 3A, B**). Furthermore, the G × E interaction was highest in E4, demonstrating that the DSR condition as the most discriminative in assessing BR and GY. PC1 contributed 87.9% and 57% of the variation for BR and GY, respectively, indicating a major contribution to the overall variation.

In the case of BR, E1 showed a strong positive association with E2 and E3 and was positioned closer to the origin, displaying PCA1 scores near zero. This suggests strong genotype performance in this environment. The RILs G3, G6, and G16 consistently excelled across environments, with stable BR values near the axis origin. RILs G5, G3, and G2 aligned closely with the average BR line and exhibited minimal environmental interaction, whereas RILs G1, G6, G8, G16, and G18 recorded higher mean BR values with strong main (additive) effects reflected by positive PC1 scores and similarly low interaction (**Figure 3A**). Thus, these genotypes can be considered ideal for improving BR.

For GY, E2 exhibited lower interaction, being closer to the origin with low PCA1 scores. E4 was a below-average environment, whereas E1, E2, and E3 had higher average yields. RIL G16 had highest mean yield, whereas four RILs–G1 and G13–G15—displayed low interaction, were placed closer to the average yield line, and remained less influenced by environmental variations. RILs G16, G13, G1, and G15, which combined high trait values with minimal interaction effects, were identified as high-yielding stable genotypes suitable for improving grain yield. RILs G3 and G5 were stable with high BR–GY across the environments, whereas RILs G1, G4, G6, and G16, with high mean values and positive interaction effects, yielded above average in both seasons, and these genotypes are best suited for favorable environments. In addition, RIL G2 was stable for BR across environments and had a high positive main effect for GY, demonstrating its adaptability and superiority for higher yield.

#### 3.4.2 Additive main effects and multiplicative interaction (AMMI 2)

AMMI2 biplots for BR (**Figure 3C**) and GY (**Figure 3D**) collectively highlight the prominent role of G × E interactions in shaping trait expression. For both traits, E4 aligned with PC1 and exhibited the longest vector, indicating high discriminating power and strong interaction effects. E1 demonstrated greater stability, indicated by its shorter vector length across both traits. RILs located near the biplot origin, including G1, G3, G5, G15, and G16, exhibited minimal interaction effects and stable performance across environments for both BR and GY. These represent promising candidates for simultaneous improvement of CS and GY. Conversely, genotypes positioned farther from the biplot origin *viz.*, G29, G27, G19, G22, and G18 for BR, and G17, G4, G23, G26, and G12 for GY, displayed increased sensitivity to environmental interactions, making them more suitable for environment-specific selection.

### 3.5 GGE biplot-based analysis

#### 3.5.1 Which-won-where pattern

The polygon view of the 3W plot, based on the inner product property of the biplot and not altered by different singular value partitioning methods vividly illustrated G × E interactions, correlating genotypes and environments along with the impact of genotype main effects for BR and GY (**Figures 3E, F**). The equality line between TC-G23 and RIL-G7 indicated that G7 performed better in E4, whereas G23 performed better in the other environments for BR (**Figure 3E**). Three RILs, G15, G1, and G8, were positioned on the line connecting G23 and G7, affirming the rank order G23 > G15 > G1≥ G8 > G7 across all environments for general adaptation. The three environments, E1, E2, and E3, were partitioned into one sector, whereas E4 was independent. G23, G15, G1, and G8 were the winners in environments E1, E2, and E3, whereas G7 was the winner in E4. This pattern suggests that the four environments can be apportioned (the target environment may consist of) into two different mega-environments and that different cultivars should be selected and deployed for transplanted and DSR conditions (**Figure 3E**). Unlike BR, the discrete classification of TP and DSR environments was not observed for GY, and all environments were partitioned into a single sector. RILs G2 and G5 are located on the line connecting RILs G16 and G4, asserting the ranking order G16 > G2 > G5 > G4 for GY (**Figure 3F**). RIL G16 emerged as the universal winner, whereas Telangana Sona, SC-G27 for BR exhibited subpar performance, particularly in E1, displaying crossover interaction.

#### 3.5.2 Mean vs. stability

**Figure 4:**
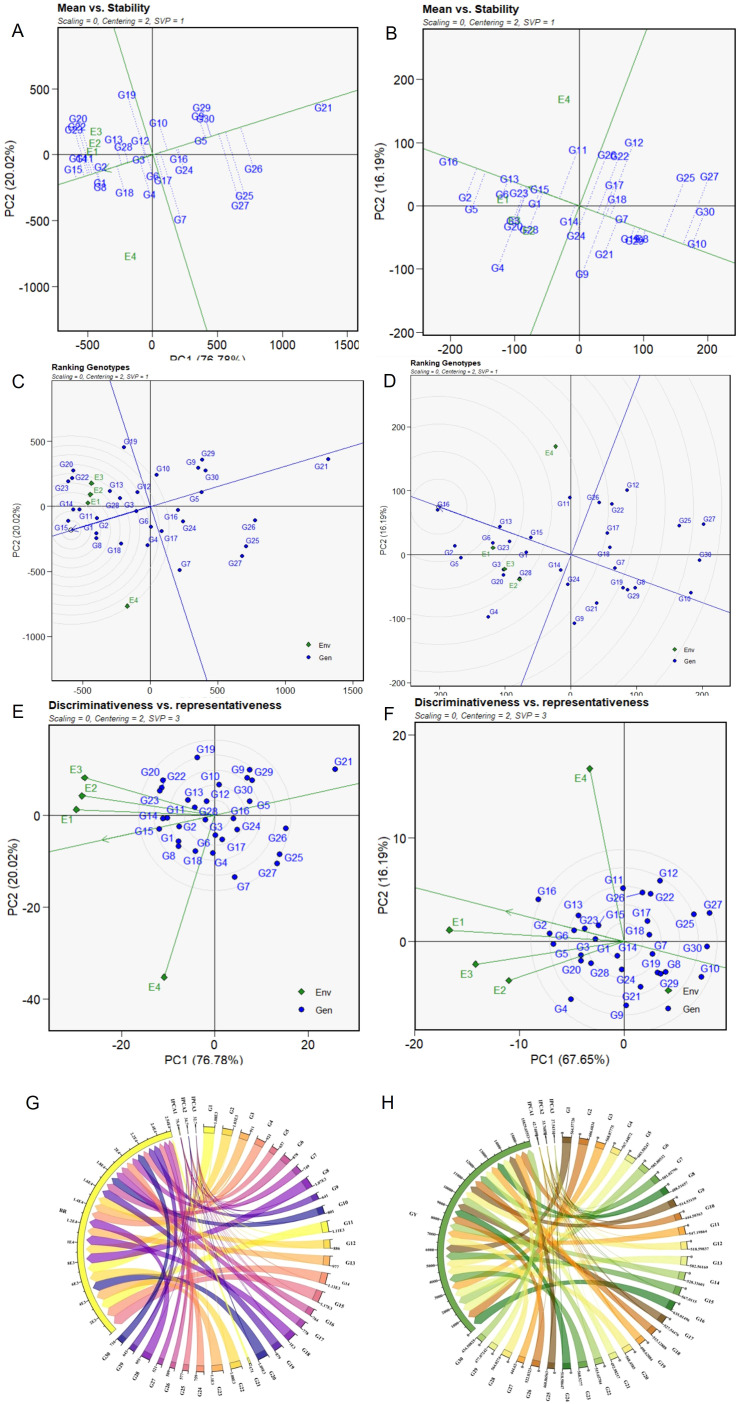
GGE biplot analysis of genotype performance and stability for breaking resistance (BR) and grain yield (GY) across four environments in 30 studied genotypes. **(A, B)** “Mean *vs*. Stability” biplots assessing both trait performance and stability of genotypes for BR and GY, **(C, D)** “Ranking Genotypes” biplots comparing genotypes against the ideal based on G + G × E interaction effects across environments for BR and GY, respectively. **(E, F)** “Discriminativeness vs. Representativeness” biplots identifying ideal genotypes and evaluating their representativeness and discriminative ability for BR and GY. **(G, H)** Circos visualization of G × E interactions for breaking resistance (BR) and grain yield (GY) in rice. Circos plot showing individual genotypes contribution to BR and GY and their respective IPCAs.

The abscissa of the AEC represents the mean trait value across environments, with higher values indicating superior performance. RILs G11, G14–15, G20 and TCs G22 and G23 exhibited the highest BR, while RILs G1, G2, and G8 closely followed (**Figure 4A**). SC-G21 demonstrated lower BR, and its plasticity stemmed from reduced BR combined with higher stability across environments. RILs G16, G2, G5, G13, G20, and TC-G23 exhibited higher GY, closely followed by G16, G1, G15, G23, and G13 (**Figure 4B**). RILs G1, G2, G15, and TC-G23 were identified as ideal genotypes, as they combined high trait expression with greater stability for both GY and CS across all four environments.

#### 3.5.3 Genotype ranking

The ranking of RILs based on mean BR follows the order G15 > G14 > G11 > G1 > G8 > G2, which stand out as the top contenders due to their close proximity to the arrowhead within the circle on the AEA (**Figure 4C**). The environment rankings, on the other hand, elucidate the sequence E1 > E2 > E3 > E4. RILs G3 and G6 assume the average genotype due to their proximity to their biplot origin, contributing minimally to G + G × E. The distance between genotypes on the biplot, corresponding to the Euclidean distance, reflected the disparities in BR and/or interactions with environments. The angle between genotypes, representing responses with proportional variations across environments, indicated similar responses (an acute angle) between RILs G1 and G2 and, likewise, an inverse relationship (an obtuse angle), implying superior performance of one genotype while the other falters, as in the case of G2 *vs*. G9, G29, and G30. In these two cases, the difference between the genotypes contribute more to G than to GE, while a right angle (e.g., G2 *vs*. G19, G2 *vs*. G7) implies independent reactions to environments, underscoring differences between them that mostly influence G × E. Also, the angle between the vector of a genotype and the AEA partitioned the vector length into components of G + G × E. A right angle (e.g., G7 to AEA) meant that the contribution was to GE only; an obtuse angle (e.g., G26 to AEA) meant that the contribution was mainly to G, which led to lower-than-average mean performance; an acute angle (e.g., G2 to AEA) reflected a contribution mainly to G, which led to higher-than-average mean performance. RILs G1, G15, G2, G11, G14, G8, and TCs-G23 and G28 outperformed in E1, E2 and E3, while RILs-G4 and G7 and breeding line G29 outperformed in E4, revealing a crossover interaction and genotype disparity for BR.

Furthermore, for GY, the genotype ranking order followed G16 > G2 > G5 > G13 > G6 > G23 > G15 > G1, while environment rankings elucidated the sequence E1 > E3 > E2 > E4 (**Figure 4D**). G16 is the ideal genotype and is more desirable, while G14 assumes the average genotype due to its proximity to the biplot origin, contributing minimally to G + G × E. The responses of genotypes RILs G1, G2, G15, G23, and TC-G28 for GY, based on the angle between, them were congruous with the responses for BR, which outperformed in E1, E3, and E2 for GY along with BR. Contrarily, G11, G26, and G22 outperformed in E4, indicating a crossover interaction.

#### 3.5.4 Discriminative vs. representative

Three environments (E1, E2, and E3) clustered together and differed from E4, indicating significant differences in both BR and GY due to the change from irrigated transplanted conditions to DSR (**Figures 4E, F**). However, the specific and general adaptation of the genotypes differed for BR and GY. For BR, the AEA traversing the biplot origin represents the average coordinates of all test environments. The first mega-environment (E1, E2, and E3) has a smaller angle with the AEA abscissa and is more representative than E4. In the first mega-environment, E1 and E2, with a subtle angle alongside the AEA abscissa, exemplify increased representativeness and discriminativeness and are advantageous for selecting generally adapted RILs (G15, G14, G23, G1, and G2), including TC G28. The wider angle observed for E4 implies reduced potential for accurate genotype discrimination. However, although discriminating but non-representative, E4 is useful for selecting specifically adapted RILs G18, G6, G3, and G4 for BR (**Figure 4E**). E1 and E3 serve as prime candidates for yield-focused genotype selection (**Figure 4F**). Conversely, E2 and E4 are considered suboptimal, suggesting limited suitability for yield. With its shorter vector, E2 displayed diminished discriminatory ability for GY. This may be due to the transplanting of older seedlings, which impacted vegetative phase duration, resulting in lower biomass and reduced GY. Our analysis of the angles between the AEA abscissa and the environment vector accentuates the differentiation of environments and shows that, for both BR and GY, E1 emerges as the optimal choice, followed by E2, E3, and E4. RILs G1, G2, and G15 and TC G23 were identified as generally adapted genotypes with high mean GY and BR. Also, RIL G16 emerged as a high yielder with optimum BR across environments.

### 3.6 BLUP-based stability statistics

In recent years, MET analysis has increasingly shifted from linear-bilinear models (e.g., AMMI), toward linear mixed-effects (LMM) models. This transition is largely driven by the fact that best linear unbiased prediction (BLUP)-based estimates of genotypic responses are more accurate than those from fixed-effect models (Piepho, 1994). Moreover, the reliability of genotype recommendations can be enhanced by jointly considering both mean performance and stability (MPS). Using BLUP, a robust methodology for estimating random effects within linear models (Smith et al., 2005), and restricted maximum likelihood (REML), several parameters—HMGV, PRGV, and HMRPGV—were estimated to measure performance and stability simultaneously (**Tables 3**, **4**). HMRPGV identified RILs G23 (0.94), G15 (0.93), G14 (−0.87), G11 (0.86), G1 (0.93), G8 (0.91), G2 (0.88), and TC G28 (0.86) as most stable and adaptable genotypes with high mean performance for BR (**Table 3**). For GY, RILs G16 (0.94), G2 (0.83), G5 (0.82), G6 (0.80), G1 (−0.81), G15 (0.80), and TC G28 (0.68) exhibited superior stable performance (**Table 4**). Considering MPS, three RILs (G1, G2, and G15) and TC G28 were identified as stable, strong-culm high-yielding genotypes across environments.

**Table 3:** Stability indices of the genotypes for breaking resistance under multi-environmental conditions for ranking of the genotypes.

GEN	BR	CV	Shukla	Ecoval	Wi_g	Pi_a	R^2^	ASTAB	ASI	ASV	AVAMGE	DA	DZ	EV	FA	MASI	MASV	SIPC	ZA	GSI	WAAS	WAASB	WAASBY	HMGV	RPGV	HMRPGV
1	1,075	5	4,290	43,190	111	16,443	0.02	8.56	2	20	183	106	0	0	11,206	2	20	3	0.07	16	3	2.29	88.83	1,069	1.27	1.26
2	1,026	11	8,077	75,005	104	24,156	0.45	39.1	2	18	311	156	0	0.04	24,382	2	18	8	0.09	36	3	2.65	84.54	1,015	1.21	1.2
3	908	5	−425	3,589	103	54,360	0.86	0.65	1	5	48	28	0	0	791	1	5	1	0.02	14	1	0.62	83.26	905	1.07	1.07
4	910	15	3,3250	286,462	71	69,938	0.53	73.97	7	59	533	309	0	0.03	95,454	7	59	10	0.21	25	8	6.72	64.06	900	1.08	1.06
5	656	6	−478	3,145	76	168,504	0.96	0.84	1	6	54	32	0	0	1,034	1	6	1	0.02	28	1	0.81	66.21	658	0.78	0.78
6	873	7	11,606	104,649	82	73,147	0.78	32.04	4	34	293	187	0	0.02	34,883	4	34	8	0.13	16	5	4.04	70.14	871	1.04	1.03
7	741	15	25,645	222,581	57	136,089	0.58	56.28	6	52	472	272	0	0.02	74,193	6	52	8	0.18	5	7	5.92	55.55	735	0.88	0.86
8	1,065	4	2,155	25,262	113	17,242	0.16	7.16	2	16	149	90	0	0	8,049	2	16	4	0.06	32	2	1.91	89.41	1,060	1.26	1.25
9	640	22	7,499	70,146	54	177,793	0.97	17.84	4	29	260	152	0	0.01	23,206	4	29	5	0.1	22	4	3.3	57.26	621	0.75	0.74
10	798	23	2,0561	179,869	65	101,324	0.67	39.72	4	37	319	205	0	0.02	42,211	4	37	9	0.14	31	5	4.61	63.42	772	0.94	0.92
11	1,107	11	3,993	40,697	120	8,482	1	10.91	3	21	183	113	0	0.01	12,719	3	21	4	0.08	45	3	2.65	89.82	1,094	1.3	1.3
12	884	14	5,742	55,389	89	63,247	0.75	10.93	2	20	178	111	0	0.01	12,392	2	20	5	0.08	16	3	2.53	75.63	873	1.04	1.04
13	975	17	13,400	119,721	92	36,144	0.81	32.66	4	35	321	192	0	0.02	37,048	4	35	8	0.13	33	5	4.32	75.93	955	1.14	1.14
14	1,130	11	4,786	47,364	122	5,938	0.98	11.53	3	23	208	122	0	0	14,962	3	23	4	0.08	35	3	2.86	90.65	1,116	1.33	1.32
15	1,065	10	5,204	50,869	125	4,595	0.67	27.38	1	10	210	122	0	0.03	14,946	1	10	6	0.06	33	2	1.65	96.74	1,153	1.37	1.37
16	760	5	3,785	38,949	77	116,552	0.01	9.98	3	22	197	114	0	0	12,947	3	22	4	0.08	30	3	2.51	67.61	761	0.9	0.9
17	778	10	6,477	61,563	77	108,359	0.03	32.96	1	6	212	129	0	0.03	16,619	1	6	6	0.04	41	1	1.03	73.47	775	0.92	0.92
18	995	11	25,924	224,920	86	42,669	0.75	87.27	5	44	420	274	0	0.06	74,926	5	44	13	0.18	57	6	5.52	73.39	987	1.18	1.16
19	879	37	81,011	687,650	46	86,012	0.77	167.01	11	91	814	470	0	0.06	221,237	11	91	14	0.31	54	11	10.24	50.83	781	1.03	0.94
20	1,085	27	59,814	509,596	81	26,248	0.93	127.29	10	79	705	410	0	0.05	168,245	10	79	12	0.27	52	10	8.99	68.27	1,025	1.27	1.23
21	169	6	1,132	16,668	19	570,376	0.95	4.56	2	13	118	73	0	0	5,298	2	13	3	0.05	20	2	2.02	30.57	179	0.21	0.21
22	1,082	27	650,12	553,263	78	29,102	0.7	162.27	10	78	715	429	0	0.08	183,830	10	78	17	0.29	45	11	9.43	66.66	1,020	1.26	1.22
23	1,094	26	59,257	504,913	82	25,011	0.85	128.78	9	78	707	409	0	0.05	167,197	9	78	13	0.27	15	10	9.04	68.72	1,035	1.28	1.24
24	754	9	14,418	128,268	66	125,031	0.69	32.78	5	40	353	207	0	0.01	42,753	5	40	7	0.14	36	5	4.53	60.79	752	0.9	0.89
25	562	47	101,275	857,869	7	271,111	0.71	216.68	12	103	926	535	0	0.08	285,842	12	103	16	0.35	36	13	11.66	25.65	521	0.68	0.61
26	499	33	45,236	387,139	21	293,044	0.64	97.9	8	69	622	359	0	0.04	129,045	8	69	11	0.24	26	9	7.97	33.24	484	0.6	0.57
27	513	28	39,248	336,843	26	280,746	0.77	84.43	8	65	577	335	0	0.03	112,077	8	65	10	0.22	33	8	7.36	36.06	502	0.62	0.59
28	949	12	4,742	46,994	98	41,539	0.82	12.96	2	20	193	115	0	0.01	13,281	2	20	5	0.08	14	3	2.59	79.69	937	1.12	1.11
29	627	44	58,921	502,095	27	202,399	0.66	286.2	5	39	697	406	1	0.27	165,157	5	39	21	0.21	44	6	5.7	48.78	576	0.74	0.69
30	713	4	4307	43,333	72	140,456	0.09	10.85	3	23	208	120	0	0	14,437	3	23	3	0.08	40	3	2.65	64.1	714	0.85	0.84

G, Represents the genotype being evaluated; GY, Mean yield of the genotype across environments; BR, Breaking resistance of the genotype across environments; CV, Coefficient of Variation; Shukla, shuklas variance; Ecovl, ecovalence; Wi_g, Weight index for genotype; Pi_a, Performance index for average yield; R^2^, Pinthus’s (1973) coefficients of determination; ASTAB, AMMI-based stability parameter; *ASI*, AMMI Stability Index; ASV, AMMI stability value; AMGE, sum across environments of genotype × environment interaction (G × I) modeled by AMMI; AVAMGE, sum across environments of the absolute value of G × I modeled by AMMI; DA, Annicchiarico’s D parameter; DZ, Zhang’s D parameter; EV, averages of the squared eigenvector values; FA, stability measure based on fitted AMMI model; MASI, Modified AMMI Stability Index; MASV, modified AMMI stability value; SIPC, sums of the absolute value of the IPC scores; Za, absolute value of the relative contribution of IPCs to the interaction; WAAS, weighted average of absolute scores; WAASB, weighted average of absolute scores for the best linear unbiased predictions (BLUPs) of the genotype-vs.-environment interaction; HMGV, harmonic mean of genotypic values; RPGV-relative performance of genotypic values; HMRPGV, harmonic mean of relative performance of genotypic values; GSI, genotype stability index; IPCA, interaction principal component axis.

**Table 4:** Stability indices of the genotypes for grain yield under multi-environmental conditions for ranking of the genotypes.

GEN	GY	CV	Shukla	Ecoval	Wi_g	Pi_a	R^2^	ASTAB	ASI	ASV	AVAMGE	DA	DZ	EV	FA	MASI	MASV	SIPC	ZA	GSI	WAAS	WAASB	WAASBY	HMGV	RPGV	HMRPGV
1	563	2.28	187	2360	102	4,189	0.02	3	0.59	2	45	28	0.11	0	787	0.6	4	3	0.05	13	0.92	0.87	77	562	1.07	1.07
2	607	7.6	3,199	27,659	97	1,341	0.55	41	1.09	4	157	96	0.45	0.07	9,220	2.03	8	9	0.16	10	2.66	2.82	72	603	1.15	1.14
3	569	5.7	1,585	14,102	96	3,352	0.24	13	2	7	122	69	0.2	0.01	4,701	2	7	5	0.13	24	2.37	2.31	65	567	1.08	1.07
4	566	13.18	7,293	62,053	82	5,168	0.46	58	4.28	14	238	144	0.4	0.05	20,684	4.28	14	9	0.25	39	4.68	4.46	43	559	1.07	1.06
5	604	5.6	1,752	15,506	101	1,299	0.31	15	2.12	7	123	72	0.2	0.01	5,169	2.12	7	5	0.13	22	2.43	2.53	74	601	1.14	1.14
6	585	3.62	907	8,413	101	2,307	0.49	13	0.89	3	95	53	0.26	0.02	2,804	0.99	5	6	0.09	11	1.56	1.66	76	583	1.11	1.11
7	496	5.98	610	5,919	86	12,594	0.27	8	0.85	3	81	44	0.18	0.01	1,973	0.87	6	5	0.08	28	1.37	1.4	51	496	0.94	0.94
8	480	10.83	2,829	24,559	75	16,418	0.01	33	1.59	5	150	90	0.37	0.05	8,186	1.62	12	8	0.14	38	2.21	2.28	37	478	0.91	0.91
9	509	9.94	3,599	31,021	79	11,235	0.39	34	2.7	9	166	102	0.35	0.04	10,340	2.72	11	10	0.21	44	3.7	3.49	34	506	0.97	0.96
10	437	12.1	2,083	18,288	68	24,103	0.52	34	0.69	2	134	78	0.43	0.06	6,096	1.01	6	7	0.1	33	1.51	1.88	27	434	0.83	0.82
11	544	11.42	2,706	23,519	87	6,863	0.89	24	2.41	8	143	89	0.27	0.03	7,840	2.41	10	7	0.17	34	3.12	2.94	51	539	1.03	1.02
12	510	17.71	6,609	56,308	72	13,124	0.93	56	3.94	13	227	137	0.42	0.06	18,769	3.95	14	11	0.26	48	4.66	4.33	27	501	0.97	0.95
13	583	4.18	603	5,860	102	2,211	0.02	10	0.4	1	72	44	0.24	0.02	1,953	0.56	4	5	0.06	6	0.95	1.16	80	581	1.1	1.1
14	528	3.84	496	4,956	93	7,229	0	5	1.1	4	79	41	0.13	0.01	1,652	1.1	5	3	0.08	22	1.44	1.34	61	528	1	1
15	564	7.77	1,276	11,509	96	4,463	0.57	20	0.62	2	106	62	0.33	0.04	3,836	0.81	6	6	0.08	13	1.27	1.04	76	561	1.07	1.06
16	635	4.08	422	4,338	114	236	0.29	5	0.77	3	65	38	0.14	0.01	1,446	0.77	5	3	0.06	6	1.09	1.47	94	632	1.2	1.2
17	517	16.26	7,131	60,690	72	12,937	0.05	71	3.21	11	263	142	0.51	0.09	20,230	3.22	18	13	0.27	44	4.7	4.47	27	508	0.98	0.96
18	507	13.98	5,370	45,898	74	13,168	0	60	2.44	8	200	124	0.49	0.08	15,299	2.48	16	12	0.23	43	3.88	3.68	32	500	0.96	0.95
19	490	10.85	2,918	25,303	76	14,884	0.01	36	1.48	5	151	92	0.4	0.05	8,434	1.55	12	9	0.13	35	2.1	2.14	42	487	0.93	0.92
20	568	6.97	2,122	18,614	94	3,608	0.15	18	2.26	8	137	79	0.23	0.02	6,205	2.26	8	6	0.15	28	2.79	2.69	61	565	1.08	1.07
21	492	6.3	1,448	12,954	82	12,375	0.22	12	1.93	6	113	66	0.19	0.01	4,318	1.93	7	5	0.12	40	2.23	2.05	43	492	0.93	0.93
22	511	13.12	3,689	31,779	79	11,310	0.49	35	2.78	9	167	103	0.36	0.04	10,593	2.82	10	8	0.18	43	3.26	3.15	38	506	0.97	0.96
23	569	7.4	1,899	16,747	94	3,170	0	22	1.47	5	139	75	0.3	0.03	5,582	1.51	9	8	0.14	17	2.4	2.39	64	566	1.08	1.07
24	519	3.98	924	8,549	89	8,525	0.63	9	1.5	5	84	53	0.17	0.01	2,850	1.51	5	4	0.09	28	1.69	1.51	57	518	0.98	0.98
25	456	16.02	4,014	34,513	67	20,395	0.9	35	2.93	10	173	107	0.33	0.04	11,504	2.93	12	8	0.21	53	3.8	3.76	15	450	0.86	0.85
26	519	13.66	3,792	32,647	80	9,931	0.85	35	2.68	9	170	104	0.34	0.04	10,882	2.68	12	8	0.2	38	3.62	3.41	38	513	0.98	0.97
27	439	17.46	4,660	39,935	62	24,176	0.76	39	3.34	11	198	115	0.34	0.04	13,312	3.35	12	9	0.22	56	4.02	3.89	8	433	0.83	0.82
28	560	6.52	2,231	19,530	92	5,051	0.66	22	1.91	6	146	81	0.28	0.03	6,510	1.91	10	7	0.16	28	2.79	2.69	59	558	1.06	1.06
29	474	8.8	1,510	13,478	78	15,466	0.15	18	1.31	4	112	67	0.28	0.03	4,493	1.34	8	7	0.13	35	2.14	1.92	39	473	0.9	0.9
30	431	10.83	1,307	11,769	70	24,344	0.85	12	1.74	6	104	63	0.19	0.01	3,923	1.74	7	5	0.13	44	2.26	2.29	21	430	0.82	0.82

G, Represents the genotype being evaluated; GY, Mean yield of the genotype across environments; BR, Breaking resistance of the genotype across environments; CV, Coefficient of Variation; Shukla, shuklas variance; Ecovl, ecovalence; Wi_g, Weight index for genotype; Pi_a, Performance index for average yield; R^2^, Pinthus’s (1973) coefficients of determination; ASTAB, AMMI-based stability parameter; *ASI*, AMMI Stability Index; ASV, AMMI stability value; AMGE, sum across environments of genotype × environment interaction (G × I) modeled by AMMI; AVAMGE, sum across environments of the absolute value of G × I modeled by AMMI; DA, Annicchiarico’s D parameter; DZ, Zhang’s D parameter; EV, averages of the squared eigenvector values; FA, stability measure based on fitted AMMI model; MASI, Modified AMMI Stability Index; MASV, modified AMMI stability value; SIPC, sums of the absolute value of the IPC scores; Za, absolute value of the relative contribution of IPCs to the interaction; WAAS, weighted average of absolute scores; WAASB, weighted average of absolute scores for the best linear unbiased predictions (BLUPs) of the genotype-vs.-environment interaction; HMGV, harmonic mean of genotypic values; RPGV-relative performance of genotypic values; HMRPGV, harmonic mean of relative performance of genotypic values; GSI, genotype stability index; IPCA, interaction principal component axis.

### 3.7 Combining BLUP and AMMI models

**Figure 5:**
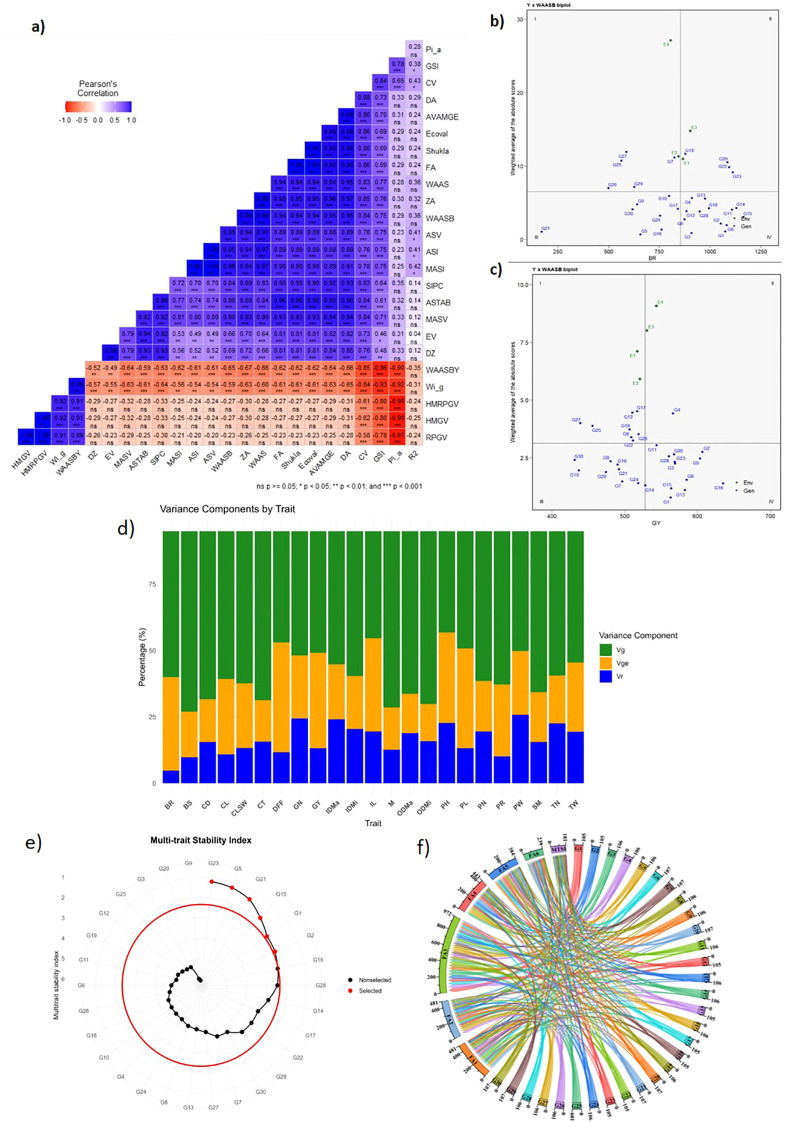
**(A)** Correlation among 24 stability indices *viz.*, HMGV, HMRPGV, ASV, ASI, MASI, MASV, Shukla’s variance, Ecovalence, CV, DA, AVAMGE, FA, WAAS index, WAASB, ZA, SIPC, ASTAB, EV, DA, CV, GSI, Pi_a_ and DZ, R^2^, **(B)** Biplot representing the relationship between BR and WAASB Scores, **(C)** GY and WAASB Scores, **(D)** Proportion of phenotypic variance for 23 culm strength and grain yield related traits, **(E)** Relative contribution of each factor on the MTSI of each genotype; **(F)** Genotype ranking and selected genotypes for the MTSI considering a selection intensity of 15%.

For the simultaneous selection of genotypes with MPS, we used a unique integrated stability parameter based on WAASB (Olivoto et al., 2019) that combines the features of both AMMI and BLUP models. The collective variance captured by the initial two IPCAs amounted to 97.3% for BR and 86.9% for GY (**Table 2**). RIL G16 emerged as the prevailing performer with a GSI of 6 (**Table 4**) across the environments, attributing to the highest yield and optimum BR (635 g/m^2^, 780 g) and lower IPCA1 scores (−1.20, 1.88) for GY and BR, respectively. Consequently, RILs G2 (1,027 g, −2.30), G11 (1,107 g, −2.85), G14 (1,130 g, −3.20), G15 (1,064 g, −1.28), G20 (1,080 g, −10.41), and TCs G22 (1,080 g, −10.31) and G23 (1,094 g, −10.33) earned the distinction of “universal winners,” with elevated mean BR values and low IPCA1 scores, indicating stability across environments (**Figure 4G**). On the other hand, RILs G2 (607 g, −3.53), G1 (563 g, −0.83), G15 (563 g, 0.07), G14 (528.2 g, −1.69), and G5 (603.58, −3.78) and TC G28 (560 g, −2.64) emerged as superior performers (**Figure 4H**). Furthermore, RILs G1 (0.77), G13 (1.09), G15 (1.17), G14 (1.19), G16 (1.27), and G23 (1.39) showcased the lowest WAASB values for GY (**Table 4**) and ranked as the most stable genotypes in the described order. Consequently, two of these RILs [G1 (1.1), G15 (1.27)] and G16 (1.34) also exhibited lower WAASB values for BR. Also, a stable and HY-RIL-G2, with lower WAASB scores for BR (2.16) and GY (1.74), respectively, was found to be promising for culm strength. This parameter is effective in identifying stable genotypes in METs. Further, parallel rankings were also observed based on other key statistics, including ASV, ASI, MASI, MASV, Shukla’s variance, Ecovalence, CV, DA, AVAMGE, FA, WAAS index, ZA, SIPC, ASTAB, EV, and DZ, and a significant positive correlation between WAASB and these indices were identified (**Figure 5A**). Additionally, a significant correlation between AMMI and BLUP stability models was observed.

WAASBY values were estimated by assigning weights of 65% to mean performance and 35% to stability (WAASB). The highest WAASBY values for BR were noted for genotypes G15 (93.9), G14 (89.1), G11 (87.4), G1 (89.2), and G2 (88.6), whereas G16 recorded 69.1 (**Table 3**). In the case of GY, the top-ranking genotypes were G16 (94.2), G13 (80.5), G1 (77.1), G15 (75.7), and G2 (72.1) (**Table 4**). To assist intuitive interpretation, a response variable *vs.* WAASB biplot was constructed (**Figures 5B, C**), which quantified stability by incorporating all IPCA values. Genotypes positioned in quadrant IV (for BR and GY) indicated high MPS, as reflected by their WAASB values. Thus, all estimated IPCA axes contributed to capturing stability in a two-dimensional framework. A parallel ranking of genotypes was observed when compared with BLUP-based stability statistics, supported by a significant positive correlation between WAASBY and BLUP-based stability measures (**Figure 5A**).

### 3.8 Multi-trait stability index

The likelihood ratio test revealed a significant G × E interaction (p <0.05) for all traits (**Table 5**), indicating a strong influence of environmental variability on genotype performance. Across all traits, the G × E variance ( $\sigma_{ge}^{2}$) was significantly higher than the genotype ( $\sigma_{ge}^{2}$) and residual variances ( $\sigma_{ge}^{2}$), indicating that genotype and G × E effects were the predominant sources of phenotypic variation (**Figure 5D**). Pearson’s correlation of WAASBY values indicated a high-magnitude correlation and grouped the traits into a common factor. Six PCs were retained by explanatory factor analysis (EFA), and the accumulated variance explained by these components was 79.99% (**Table 5**). After varimax rotation, the average communality (*h*) was 0.89 and WAASBY values for the 23 traits were grouped into six factors (FA) (**Table 5**). FA 1 primarily represented a composite dimension integrating structural support traits such as PR, BS, and DFF, which reflect crop duration and adaptation. FA 2 represented the core structural and yield accumulation traits and served as a critical interface between lodging resistance and grain yield. PH, IL, and CL determine the leverage force acting on the plant, while CD, CT, SM, M, and BR contribute to stem strength and lodging resistance. GN and GY indicate assimilate production and partitioning. This factor reflects the critical balance between increased yield potential and structural stability, making it central to the simultaneous improvement of lodging resistance and grain yield. Selection within this factor can optimize plant architecture to enhance grain yield while improving lodging resistance. FA 3 and FA 4 encompassed traits associated with ODMi, ODMa, IDMa, IDMi, TN, PN, and CLSW. These traits collectively enhance stem solidity and anchorage, thereby improving lodging resistance. FA 5 and FA 6 grouped grain yield-related traits, including PL, PW, and TW, which are indicative of grain-filling efficiency and yield quality. Selection for mean performance and stability across multiple traits was based on genotype–ideotype (Euclidean) distance using scores obtained from an EFA, and the scores for the 30 genotypes across the first six factors are presented in **Supplementary Table 4**.

**Table 5:** Summary of the results for random- and fixed-effects, eigen values, explained variance, factorial loadings after varimax rotation, communalities obtained in the factor analysis and selection differential of the WAASBY index for 23 strong culm related traits.

Trait	LRTg	LRTge	FA1	FA2	FA3	FA4	FA5	FA6	Communality	Uniqueness	Xo	Xs	SD	SDperc
PH	3.38E−09	3.64E−25	−0.27	0.33	−0.16	−0.17	−0.01	−**0.73**	0.77	0.23	51.56	75.16	23.59	45.76
IL	2.35E−09	2.58E−30	0.28	−0.09	0.14	0.17	−0.02	−**0.85**	0.86	0.14	54.08	56.82	2.74	5.07
CL	7.88E−15	2.36E−40	−0.32	**0.75**	−0.10	−0.08	−0.03	−0.45	0.89	0.11	58.76	80.50	21.74	37.00
TN	3.49E−17	1.11E−13	0.55	−0.05	**0.62**	−0.42	0.14	0.07	0.88	0.12	49.65	50.68	1.03	2.08
PR	1.01E−15	2.61E−41	−0.11	**0.88**	0.11	0.01	−0.08	−0.08	0.82	0.18	53.85	60.46	6.60	12.26
CD	1.96E−21	1.2E−17	−**0.85**	0.37	−0.05	0.10	−0.08	−0.07	0.89	0.11	68.12	81.43	13.31	19.54
CT	7.9E−22	6.45E−17	−**0.82**	0.13	−0.36	0.01	−0.10	0.00	0.83	0.17	61.50	73.80	12.29	19.99
SM	2.05E−19	2.33E−20	−**0.88**	0.26	−0.13	−0.01	−0.16	−0.13	0.90	0.10	58.80	72.91	14.11	24.00
BS	6.17E−23	9.19E−29	−0.29	**0.82**	−0.22	0.07	0.01	−0.15	0.84	0.16	57.05	64.42	7.37	12.92
M	8.42E−23	2.46E−21	−0.59	**0.66**	−0.08	0.01	−0.05	−0.20	0.83	0.17	52.59	70.26	17.68	33.61
BR	1.21E−15	8.33E−31	−0.45	**0.67**	0.17	0.03	−0.04	−0.05	0.68	0.32	66.64	84.71	18.06	27.11
CLSW	2.2E−16	3.28E−30	0.54	−0.12	−0.11	0.04	**0.57**	0.29	0.73	0.27	39.88	53.20	13.32	33.41
DFF	3.36E−09	6.85E−50	0.01	**0.73**	0.17	0.10	−0.06	0.06	0.58	0.42	52.53	64.48	11.95	22.76
PN	1.58E−17	5.57E−17	**0.56**	0.00	0.27	−0.42	0.23	−0.08	0.63	0.37	39.08	41.66	2.58	6.59
PL	3.19E−10	1.41E−42	0.05	−0.30	0.12	−0.45	−0.35	**0.57**	0.76	0.24	57.45	58.11	0.66	1.14
GN	3.12E−13	1.02E−16	0.12	−0.07	0.19	−0.24	**0.85**	−0.15	0.85	0.15	58.07	71.08	13.01	22.40
PW	1.07E−12	7.05E−16	0.01	0.17	**0.84**	0.19	0.07	0.01	0.78	0.22	52.68	55.55	2.87	5.45
TW	1.37E−13	8.56E−23	−0.16	0.10	0.18	**0.77**	−0.20	−0.11	0.71	0.29	50.50	55.11	4.61	9.13
GY	1.07E−14	2E−49	−0.24	**0.58**	0.28	0.36	0.13	0.11	0.63	0.37	49.63	72.22	22.59	45.53
Eigen values			9.08	3.04	2.06	1.45	1.28	1.12	–					
Variance %			39.50	13.21	8.96	6.31	5.57	4.87	–					
Cumulative %			39.50	52.71	61.68	67.99	73.55	78.42	0.78					

Significant at P <0.001; LRTg and LRTge, Likelihood ratio tests for genotype and interaction G × E. PH, plant height; IL, internode length; CL, culm length; TN, tiller number; PR, pushing resistance; CD, culm diameter; CT, culm thickness; SM, section modulus; BS, bending stress; M, bending moment at breaking; BR, breaking resistance; CLSW, culm with leaf sheath weight; DFF, days to 50% flowering; PN, panicle number; PL, panicle length; GN, grain number; PW, panicle weight; TW, test weight; GY, grain yield; FA factor retained; Bold values indicate variables grouped within each factor. (*Xo*), original average for WAASBY index; (*Xs*), mean for WAASBY index of the selected genotypes (G23, G1, G2, G15); SD, selection differential; SD perc, selection differential percentage. Bold values represent the traits grouped in the factor (FA).

#### 3.8.1 MTSI and genotype selection

Assuming a selection intensity of 15% and a threshold MTSI value of 2.36, seven stable and adaptable genotypes—G23, G5, G21, G15, G1, G2, and G16—were identified, with MTSI values of 1.19, 1.27, 1.43, 1.86, 2.21, 2.23, and 2.36, respectively (**Figure 5E**). G28 was positioned near the selection threshold boundary, indicating its potential relevance for further evaluation. The contribution of each factor to the MTSI revealed that approximately 33.6% of the distance from G1 to the ideotype was attributable to FA5, and approximately 23.6% was related to FA4 (**Figure 5F**). This indicates that G1 expressed lower WAASBY values for PL (FA3) and for TN and PN (FA4). FA2 contributed only 1.5% to the MTSI for G1, suggesting close alignment with the ideotype for CS and GY traits, which are critical for grain yield with improved lodging resistance. For G2 and G15, FA4 accounted for the largest share of the MTSI (approximately 21.8% and 29.5%, respectively). This suggests that a breeding program should aim to improve TN and PN for these genotypes, while FA2 has a low contribution of only 4.9% to the MTSI, indicating their proximity to the ideotype for BR and GY. For TC G23, FA2 contributed 8.6% to the MTSI, indicating proximity to the ideotype for CT, SM, and CD, and the breeding program should be directed toward improving TN, PN (FA3), and DFF (FA1).

The selection differential for the WAASBY index was positive for most traits. The mean of the selected genotypes (*Xs*) was higher than the original average (*Xo*), suggesting that the method was efficient in selecting the best-performing and stable genotypes. The highest selection differentials (SD) was for GY (44.65%), M (29.97%), PR (27.62%), and BR (26.57%) (**Table 5**). The magnitude of the SD across traits clearly indicates how strongly selection discriminated superior genotypes and which traits offered the greatest exploitable variability. Strong culm traits such as M, SM, and BR exhibited greater selection differentials, demonstrating that the selected genotypes (G23, G1, G15, G2, G5, and G16) were markedly superior for stem structural strength, overall plant mass, and breaking resistance. Application of the WAASBY–MTSI resulted in clear selection responses across the evaluated traits. Positive selection differentials and selection gains, supported by consistently high heritability estimates (h² = 0.78–0.94), were observed for yield-related and CS traits, including GN (SG% = 17.50), GY (SG% = 5.35), BR (SG% = 15.58), and M (44.96%), indicating that the index efficiently captured genetic variability associated with enhanced culm strength and assimilate production. Moderate to high gains were also recorded for structural and physiological traits such as SM, CL, CD, CT, PN, TW, PW, and CLSW, demonstrating simultaneous improvement of multiple interrelated traits (**Supplementary Table 5**). Importantly, IL (SG% = −10.82) and DFF (SG% = −3.98) represent favorable outcomes. Shorter basal internodes contribute to improved lodging resistance and structural stability, while earlier flowering enhances crop adaptation by facilitating stress escape under variable or marginal environments. The relatively high heritability associated with these traits (h² ≥0.83) indicates that these desirable reductions are expected to be reliably transmitted to subsequent selection cycles. Likewise, modest gains in PH, PL, PR, BS, and TN reflect balanced plant architectural optimization rather than extreme directional change, reinforcing the ideotype-driven nature of the selection process.

### 3.9 Genotype stability index

GSI, a non-parametric method, is increasingly used for the simultaneous selection of MPS. Lower GSI scores for G3 (14), G13 (14), and G28 (14) indicated higher BR with high stability, followed by G23 (15), G1 (16), G6 (16), and G12 (16) (**Table 3**). The same genotypes were in quadrant IV of **Figures 5A**, characterizing them as highly stable. G16 and G13, with low GSI values (6), outperformed all the genotypes for GY. G6 (11), G1 (13), and G23 (17) displayed stability for both GY and BR (**Table 4**).

The combined application of AMMI, GGE biplot, BLUP-based stability statistics, and multi-trait indices consistently confirmed significant G × E interactions, enabling the precise identification of stable and high-performing genotypes. Across analytical frameworks, RIL 417 (G1), RIL 419 (G2), RIL 314 (G15), and RIL 315 (G16), along with the strong-culm check IRGC 10658 (G23), repeatedly demonstrated strong culm traits with high grain yield and broad adaptability. Among environments, DSR emerged as the most informative for differentiating genotypic responses for both CS and GY traits. BLUP and WAASB analyses enhanced reliability by combining mean performance with stability, while MTSI and GSI provided robust criteria for multi-trait selection. Collectively, these integrative analyses identified and validated a set of RILs suitable for the simultaneous enhancement of culm strength and yield across diverse rice-growing environments.

## 4 Discussion

Lodging is one of the most critical constraints in rice cultivation, often resulting in severe yield losses and deterioration of grain quality, with reductions reported between 10% and 40%, depending on crop stage and severity (Berry et al., 2004). Although semi-dwarf varieties have revolutionized rice yields, their altered plant architecture (Liu et al., 2018), thinner culms, and shallow root systems frequently predispose them to lodging, especially under unfavorable weather events such as heavy rainfall or strong winds (Kashiwagi et al., 2008). While agronomic measures such as regulating planting density (Lang et al., 2012; Li et al., 2011; Mobasser et al., 2009), optimizing nitrogen inputs (Zhang et al., 2014), or applying growth regulators (Okuno et al., 2014) can help reduce lodging incidence, genetic improvement remains the most reliable and sustainable strategy. Breeding efforts for lodging resistance have targeted enhancing stem strength (Ookawa et al., 2010; Badri et al., 2024) increasing culm diameter (Zhao et al., 2025; Mamidi et al., 2025), improving lignin deposition (Liu et al., 2018; Chen et al., 2011), and strengthening root anchorage (Kang et al., 1999), without compromising yield potential (Li et al., 2020). For rice varieties to achieve widespread acceptance among farmers, it is essential to develop reliable, climate-resilient, and well-adapted genotypes evaluated through rigorous multi-environment trials (METs). Addressing G × E complexity using appropriate statistical tools is the most effective way to identify adaptable and stable, high-yielding strong-culm genotypes for the deployment of climate-resilient rice in the targeted environments. Although multiple studies involving stability analysis have been documented for various traits in rice, to the best of our knowledge, this study reports for the first time G × E interaction analyses of culm strength and lodging resistance in rice.

There have been multiple studies involving stability analysis using different G × E datasets. The mean value of a trait may suffice to evaluate the stability of a genotype when G × E interactions are negligible (Yan and Kang, 2002). However, stability models are primarily aimed at simultaneously considering both genotypic main effects and G × E interactions in relation to mega-environments and test sites (Yan et al., 2007). Multi-season trials often reveal crossover G × E interactions, where genotypic rankings vary across environments, and non-crossover G × E interactions, where rankings remain constant. Tools such as AMMI and GGE biplots are widely employed to visualize and dissect these interactions, and have proven effective in evaluating yield stability and stress tolerance in rice (Panda et al., 2023). Among these, AMMI is generally regarded as more reliable, or at least comparable, to GGE (Gauch, 2006). More recently, MTSI (Olivoto et al., 2019) has gained importance because it simultaneously accounts for multiple traits in selection, unlike traditional models that focus on one trait at a time. Under high G × E conditions, robust approaches integrating WAASB and MTSI can significantly improve selection efficiency (Abdelghany et al., 2021). Therefore, evaluating lodging resistance through multi-environment trials using advanced stability models provides a comprehensive understanding of genotypic performance and helps identify cultivars that combine structural resilience with yield stability across environments.

The normal distribution observed for culm strength (CS) and grain yield (GY) in the present study highlights the wide variability in trait expression among the evaluated genotypes, providing an ideal basis for genetic improvement. The reduced grain yield under DSR conditions was primarily associated with earlier flowering and lower values for key yield-contributing traits such TN, PN, TW, PW, and GN compared with transplanted environments. This reflects established patterns associated the known challenges of DSR, where environmental stresses and altered resource availability often constrain yield potential relative to conventional transplanted systems (Xu et al., 2019, 2022).

Notably, eight RILs (G1–G3, G8, G11, G12, G14, and G15) exhibited culm diameters more than 3.2-fold thicker and over 5-fold wider than the lodging-sensitive checks (SC-G21 and G27) and more than 1.5 times thicker than the lodging-tolerant check (TC-G26). These superior structural traits could be attributed to the presence of favorable alleles of key CS-QTLs such as qCT7 (Chr 7, 109 kb, PVE: 14.49%), *qCD3* (Chr 3, 122 kb, PVE: 8.39%), *qSM8* (Chr 8, 32 kb, PVE: 12.05%), and qIBW7 (Chr 7, 33kb, PVE: 16.57%) associated with breaking resistance identified in these lines (Mamidi et al., 2025). Similarly, the remarkable culm thickness observed in RILs G1 and G4 (up to 2.82 mm) could be attributed to the presence of favorable alleles of *qCT7*, while three wider-culm RILs, G5, G16, and G18, possess the *qCD3* allele associated with enhanced culm diameter. The presence of *qSM8* in RILs such as G1 and G19 further contributed to increased section modulus values, a critical determinant of mechanical strength. Additionally, RILs carrying favorable alleles of *qIBW7* (e.g., G11, G12, G13, G14, and G17) demonstrated high resistance to culm breaking, further supporting the functional role of these QTLs in enhancing structural integrity. These results collectively reinforce the potential of these QTLs for improving culm architecture. In particularly, *qCT7*, *qCD3*, *qSM8*, and *qIBW7*, can significantly enhance lodging resistance by reinforcing stem robustness, thereby offering a targeted breeding strategy for culm structural resilience in rice. The functional efficacy of these QTLs may be attributed to the presence of candidate genes such as *SAMSL* (Wu et al., 2022), which regulates lignin deposition, and *OsCesA3* and *OsCesA6*, which are involved in cellulose biosynthesis (Zhou et al., 2024). Supporting genes such as *OsBC1L7* (a COBRA-like protein), *CLD1*, and *OSH15* further enhance cell wall integrity and plant stature (Dai et al., 2009; Xiang et al., 2012). The coordinated expression of these genes strengthens secondary cell wall formation, thereby improving mechanical strength and contributing to lodging resistance. In parallel, the presence of *qSPY5*, a grain yield QTL, in eight RILs (G5, G7, G10, G15, G17, G18, G19, and G20) appears to complement the mechanical strength conferred by culm-related QTLs. These lines maintained strong culms and produced high grain yield under varying conditions, suggesting a synergistic relationship between CS and GY traits. Thus, the presence of both culm structural and yield-related QTLs in these RILs underscores their potential as donors for breeding high-yielding, lodging-resilient rice cultivars.

The current study clarified the component traits associated with GY-BR within the developed RILs. Despite the tall stature and heavy panicles in donor parent, IRGC39111 and RILs, their lodging resistance, could be attributed to higher BR (760 g–1,130 g), wider culms (5.25 mm–7.78 mm; **Figure 2F**) and thicker culms (1 mm–2.82 mm). Consistent with our findings, a high positive correlation was observed between PH and biomass (Niklas and Enquist, 2001) and BR (Nomura et al., 2019). Long-culm varieties have higher CO_2_ diffusion efficiency in the canopy due to lower leaf area density compared with short-culm varieties, thereby promoting biomass production (Nomura et al., 2019). This was supported by the higher culm-with-leaf-sheath weight (CLSW) (8.70 g–14.15 g) observed in the RILs, suggesting that during the growth period, canopy structure and CO_2_ diffusion were maintained, resulting in higher CLSWs. Further investigation of canopy structure, leaf area density, and CO_2_ diffusion within the canopy is warranted. In our study, we observed sub-species differences in CS traits. Both culm morphological and physical traits exhibited strong positive associations among themselves (Badri et al., 2024). Notably, a strong positive association between physical strength traits and GY (Guo et al., 2021; Badri et al., 2024) was observed, suggesting that improvements in CSLW lead to increases in PW and, ultimately GY. A trade-off was observed between TN and physical strength traits, indicating a compensation of number for strength (Hirano et al., 2014; Chigira et al., 2023; Badri et al., 2024; Mullangie et al., 2024). The rice crop is constantly subjected to self-weight-generated-BM during its maturity as the growing panicle becomes increasingly heavy during maturity, as the growing panicle becomes increasingly heavy, causing the whole plant to bend downward. However, we observed that RILs (**Figure 2E**) with increased BR, due to the increased CT (**Figure 2G**), exhibit long and heavy panicles that counterbalance the BM. This observation is consistent with a previous study by Ma et al. (2004). Improving BR is the most effective measure to enhance BR, thereby bolstering rice GY and its components. Culm stiffness, expressed as BS, is an intricate product of structural carbohydrates (CHOs; lignin and cellulose) and non-structural-CHO contents of the lower internode. CHOs in culms accumulate before heading and are transported to the panicles, with non-structural CHOs primarily transported to grains for grain filling. Thus, culm stiffness increases with higher structural CHO content in the basal stems (Zhang et al., 2014). This necessitates the assessment of the role of structural CHOs in culm stiffness in future research. Additionally, PL was observed to have a stronger correlation with physical strength and CT than CD, while Ookawa et al. (2010) reported a strong positive association between wider culms and GN. Furthermore, PW is positively associated with physical strength traits and thicker culms, while GN is associated with wider culms, offering scope to explore the physiological pathways underlying the partitioning of photo-assimilates into various yield component traits relative to culm strength traits.

The integration of ANOVA and PCA provided valuable insights into the substantial impacts of location, genotype, and their interactions on CS and GY. In our comprehensive analysis, which considered for two PCs, the explanatory power reached 97.3% and 86.9% of the variability, surpassing the commonly accepted minimum threshold of 70% and demonstrating significant reliability. We observed that increased temperature reduced both BR and M, resulting in weaker culms. The decline in M was linked to a reduction in SM, which in turn was driven by decreased CD and CT. Additionally, CL increased significantly with higher temperatures, potentially contributing to the decline in BR. These findings are consistent with previous reports by Luo et al. (2022). Multi-environment data may exhibit crossover G × E interactions, where genotype rankings shift across environments; in this study, the observed G × E was of the qualitative (crossover) type. Our investigation employed multiple models—emphasizing AMMI, GGE, BLUPs, and MTSI—to partition and interpret the data, thereby elucidating the intricate dynamics of rice genotypes and their stability and versatility for CS and GY traits.

The AMMI model explored multi-directional aspects of G × E interaction, enhancing the reliability of genotype evaluation across environments. The significant sum of squares for genotypes reflected inherent genetic differences, accounting for variability in CS and GY. Significant variation among environmental effects confirmed that each environment differed distinctly from the others. The large sum of squares for G × E interactions indicated a significant influence of the interaction between genotypes and environments. AMMI1 provided detailed insights into G × E interactions and facilitated the identification of genotypes that excelled in specific environments as well as those displaying stability across multiple environments. AMMI1 clearly illustrated the dominance of environment effects over genotype effect, as evidenced by long environmental vectors and genotypes clustered near the origin. Genotypes positioned farther from the origin—G26, G25, G27, and G5 for BR, and G10, G4, and G9 for GY—exhibited strong interactions with specific environments, as indicated by both positive and negative IPCA1 scores (**Figures 3E, F**). Significant environmental differences highlighted genotypic variation in adaptability, and genotypes varied considerably in their stability for yield traits, consistent with earlier observations by Balakrishnan et al. (2016) and Zewdu et al. (2020). The AMMI model effectively partitioned G × E interactions and identified genotypes (G1, G3, G6, G15, G16, and G28) with both high mean performance and stability for CS and GY.

AMMI2, based on PC1 and PC2 scores, provides a distinct advantage by offering a concise overview of complex G × E interactions across multiple factors. The interaction of 30 genotypes across four environments are captured by the first two PCs for both BR and GY. Thus, RILs G1, G3, G5, G13, G15, and G16, positioned near the origin and exhibiting high BR and GY values compared to the checks, demonstrate general adaptation across environments and consistent responses, and can be regarded as stable and useful genotypes (**Figures 5C, D**). Higher BR and CLSW, which promote greater biomass, may enhance the yield of rice genotypes. GGE biplots for BR and GY demonstrated their potential usefulness in identifying superior genotypes across environments. Environmental differences, together with genetic variation among the tested genotypes, ultimately generated variable genotypic responses. The first two PCs in GGE biplots explained approximately 96.8% of the variation in BR and 93.84% in GY. GGE enabled the delineation of mega-environments and evaluation of cultivar stability across diverse cropping systems (Mohammadi et al., 2012; Rakshit et al., 2012; Amiri et al., 2015). Nassir (2013) investigated the stability and adaptability of upland rice genotypes using both predicted genotypic and phenotypic values. A key advantage of this approach is its ability to facilitate simultaneous selection for high mean performance and stability across multiple environments, compared to AMMI (Yan et al., 2000). This enables the identification of superior genotypes with minimal stability variance, ultimately improving selection efficiency.

In the present study, the *which-won-where* polygon was employed to select diverse genotypes for BR and GY for a specific environment. The ranking for BR across all environments was G23 > G15 > G1 ≥ G8 > G7, whereas for GY it was G16 > G2 > G5 > G4. These rankings, based on the inner-product property of the biplot, remain consistent regardless of the singular value partitioning method used. Mean-versus-stability plots showed stable, high GY combined with greater CS across environments for RILs G1, G2, and G15, as well as the TC G23. Interestingly, G1 and G23 also exhibited high GN with greater stability (**Supplementary Figure 1**), consistent with the synergistic association between wider culms and GN reported by Ookawa et al. (2010). Consistent with the reported positive association between PW and CS (Guo et al., 2021; Badri et al., 2024), G2 as an ideal genotype for PW (**Supplementary Figure 1**). Among the four environments, E4 (DSR) was the most discriminative. Furthermore, the genotype-ranking biplot provides critical insights into variations in genotype performance across environments, taking both mean performance and G × E interactions.

BLUP outperforms any member of the AMMI family in predicting yield within the context of METs, as it accounts for intrinsic factors specific to each trial Piepho (1994). Therefore, a judicious choice between AMMI models and BLUP is crucial for accurate performance prediction, and our results align with Piepho’s findings. Parametric and non-parametric methods, including AMMI and GGE biplots, assess stability and mean performance on a trait-by-trait basis. For simultaneous selection based on mean performance and stability across multiple traits, we employed WAASB and WAASBY indices (Olivoto et al., 2019). The WAASBY biplot divided the genotypes into four quadrants, with the stable genotypes positioned in quadrant IV, confirming their superiority for both BR and GY. The positive SD% of WAASBY scores for all the studied traits indicated that the method efficiently selected the best-performing and superior genotypes. The correlation among all the statistical measures provided valuable insights, offering breeders guidance in selecting the appropriate model for METs. A key advantage of MTSI is its ability to fine-tune weights and rescale traits according to the breeder’s priorities (Singamsetti et al., 2021). MTSI is calculated based on the genotype–ideotype distance derived from factor analysis scores. MTSI identified the most stable genotypes exhibiting high BR and GY. Four top entries were selected with 15% SI based on their low MTSI values. All genotypes selected through MTSI were also located closer to the AEC in the mean-versus-stability biplot. The relative contribution of each factor in the MTSI highlighted the target traits that should be improved to achieve stability. Globally, numerous studies have investigated G × E studies for grain yield. However, to date, no study has reported on strong culm traits. Here, we report for the first time a multi model-based G × E study simultaneously evaluating culm strength and grain yield traits.

The integrated use of QTL information with G×E interaction analysis through AMMI and GGE has proven effective in identifying elite RILs that are both genetically superior and phenotypically stable. Importantly, the stability of these QTL-associated lines reinforces the value of combining molecular selection with phenotypic multi-environment trials. The synergy between QTL identity and phenotypic adaptability, as revealed through AMMI and GGE, provides a strategic solution for breeders. This ensures that selected genotypes are not only genetically endowed but also environmentally competent, thereby increasing the likelihood of success in both general and specific adaptation breeding programs. However, wide segregation—even in the advanced generations—is expected for adaptive traits, resulting in pronounced G × E interactions in lines derived from *indica* × tropical *japonica* cross. Unstable RILs, such as G6, G13, and G18 across E1–E4, likely represent continued segregation in advanced generations. The observed crossover interaction patterns highlight how *japonica* introgressions contributed to increased variability in performance within an *indica* background across environments. Assessment of trait consistency across environments in *indica* and tropical *japonica* derivatives therefore necessitates stability analysis. G × E interaction studies enabled the identification of stable RILs with reliable performance. While instability is undesirable for traits such as yield and culm strength stability, it provides opportunities to exploit environment-specific adaptation. Stable lines serve as strong candidates for multilocation trials, whereas unstable but high-performing genotypes offer promise for further breeding or QTL mapping to harness specific adaptive alleles.

## 5 Conclusion

• The present study highlights a multi-model statistical approach for assessing stable introgressions of superior culm strength and grain yield traits from tropical *japonica* (IRGC39111) into the elite *indica* (Swarna) background.
• By employing a range of stability metrics and considering rankings from multiple approaches, three RILs [RIL 417 (G1), RIL 419 (G2), RIL 314 (G15)] and one tropical *japonica* accession, IRGC 10658 (TC-G23), exhibited superior performance, adaptability, and stability for grain yield and lodging resistance across environments.
• The study demonstrates that BLUP-based statistics provide a reliable method for identifying stable and superior genotypes across diverse environments. By considering variance across environments and treating genotypes, environments and interaction as random effects, BLUP reduces bias in performance estimation thereby facilitating the development of superior cultivars with broad adaptation
• Integrating QTL-informed selection with multiple stability models enabled a comprehensive evaluation of the genetic potential and environmental adaptability of the RILs.
• This study provides a solid foundation for the breeding of these selected genotypes toward climate-resilient rice varieties capable of withstanding diverse environmental challenges.

## Data Availability

The original contributions presented in the study are included in the article/**Supplementary Material**. Further inquiries can be directed to the corresponding author.

## Supplementary material

Supplementary Figure 1 Representation of Mean *vs*. Stability GGE biplot for **(A)** Grain number, **(B)** Panicle length, **(C)** Panicle weight, **(D)** Test weight.
