# Supplementary figures and images for: A multi-model genotype × environment interaction analysis discerning phenotypic plasticity of the strong culm trait in rice
Source: Front Plant Sci. 2026 May 13;17:1727579. doi: 10.3389/fpls.2026.1727579 (PMC13212538; doi:10.3389/fpls.2026.1727579)

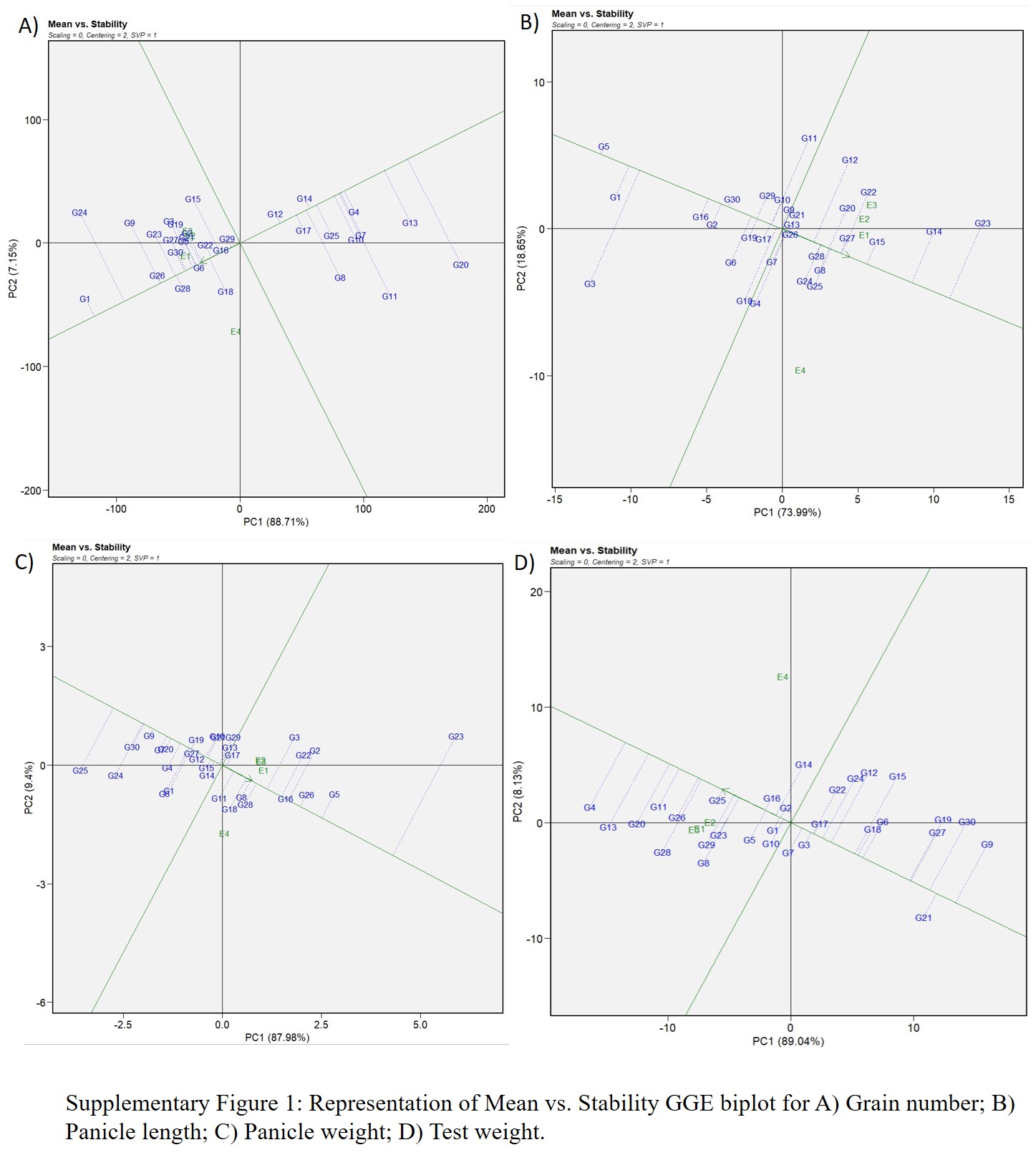

Supplement: Supplementary Figure 1 — Representation of Mean vs. Stability GGE biplot for (A) Grain number, (B) Panicle length, (C) Panicle weight, (D) Test weight. [file Image1.jpeg]
