# Supplementary material for: A multi-model genotype × environment interaction analysis discerning phenotypic plasticity of the strong culm trait in rice
Source: Front Plant Sci. 2026 May 13;17:1727579. doi: 10.3389/fpls.2026.1727579 (PMC13212538; doi:10.3389/fpls.2026.1727579)
Supplement: Supplementary file 2 [file Table1.docx]

**Supplementary Table S1. Description of genotypes used in the study.**

| **Code** | **Genotype** | **Pedigree** | **Description** |
| --- | --- | --- | --- |
| **G1** | RP 6717-891-RIL-417 | Swarna / IRGC 39111 [high grain yield (400-650 g/m^2^) and Breaking resistance (700-1400g )] | Possess *qCT7* (Chr 7, 109kb, PVE:14.49%) (G1:1.91 mm, G2:2.82 mm; G3: 2.22 mm; G4:1.92 mm) for culm thickness; *qSM8* **(Chr 8, 32kb, 12.05%)** (G1:29.48mm^3^, G2:32.25mm^3^, G3:23.40mm^3^, G4:18.45mm^3^) for section modulus |
| **G2** | RP 6717-891-RIL-419 |  |  |
| **G3** | RP 6717-891-RIL-421 |  |  |
| **G4** | RP 6717-891-RIL-424 |  |  |
| **G5** | RP 6717-891-RIL-428 |  | Possess *qCT7* (1.68 mm), *qCD3*(5.79 mm), *qSPY5* |
| **G6** | RP 6717-891-RIL-34 |  | Possess *qCT7* (1.51mm) for culm thickness |
| **G7** | RP 6717-891-RIL-147 |  | Harbour *qSPY5* (Chr 5, PVE: 11.58%) |
| **G8** | RP 6717891-RIL-181 |  | Possess *qCT7* (G8-2.27, G9-1.92) for culm thickness |
| **G9** | RP 6717-891-RIL-198 |  |  |
| **G10** | RP 6717-891-RIL-214 |  | Harbour *qSPY5* |
| **G11** | RP 6717-891-RIL-222 |  | Harbour *qIBW7* **(Chr 7, 33kb, 16.57%)** (1107g) |
| **G12** | RP 6717-891-RIL-246 |  | Harbour *qSM8* (CT: 2.47mm)*;* *qIBW7* |
| **G13** | RP 6717-891-RIL-297 |  | Harbour *qCT7* (2.18mm), *qIBW7* |
| **G14** | RP 6717-891-RIL-310 |  | Harbour *qCT7* (2.65 mm), *qIBW7* |
| **G15** | RP 6717-891-RIL-314 |  | Harbour *qSPY5* |
| **G16** | RP 6717-891-RIL-315 |  | Harbour *qCD3* **(Chr 3, 122 kb, PVE:8.39%),** (5.25mm) |
| **G17** | RP 6717-891-RIL-340 |  | Harbour *qIBW7*, *qSPY5* |
| **G18** | RP 6717891-RIL-346 |  | Possess *qCD3* (6.12mm), *qSPY5* |
| **G19** | RP 6717-891-RIL-348 |  | Possess *qSM8* (20.17mm3), *qSPY5* |
| **G20** | RP 6717-891-RIL-352 |  | Possess *qCT7* (1.82mm), *qSPY5* |
| **G21** | Swarna | Vasistha/Mahsuri | Elite *indica* cultivar with high adaptability, low nitrogen responsive, popular mega-variety with stable yield and lodging sensitive. |
| **G22** | IRGC 39111 | Accession | Tropical *japonica* accession with strong culm. Found to be a novel source of strong culm deviod of *SCM2.* Registered as strong culm donor with NBPGR- IC no: 650729 (Badri *et al.* 2018; Bagudam *et al.* 2020; Badri *et al.* 2023) |
| **G23** | IRGC 10658 | Accession | Tropical *japonica* strong culm accession (Kamala, 2024) |
| **G24** | RMS 2085 | (Improved samba mahsuri/Habataki// ST 12 | High yielding and strong culm introgression line possessing *SCM2, OsSPL14, Gn1a*. (Koushik *et al.* 2024 ). |
| **G25** | Tellahamsa | HR12/TN1 | *Indica*; Lodging sensitive check; Tolerant to cold |
| **G26** | JGL-24423  (Jagitial Rice-1) | MTU 1010/NLR 34449//MTU 1010 | *Indica*; A high yielding strong culm cultivar. Regional check for lodging tolerance |
| **G27** | RNR-15048 (Telangana sona) | MTU 1010/JGL 3855 | *Indica*; Popular variety in Telangana known for its high yields and low GI. Lodging sensitive check |
| **G28** | RNR-28361 | Bhadrakali/HKR 05-22 | *Indica*; A medium duration long slender variety with strong culm. Suitable for export with more grain length. Lodging tolerant check |
| **G29** | RNR-29325 | TME 80518 /BPT 5204 | *Indica*; A short duration long slender variety. Moderately resistant to BPH and leaf blast with high Head Rice Recovery (62.1 %). |
| **G30** | RNR-31479 | KMP 199 / RNR 15048 | *Indica*; Short duration culture with short slender grain. |

**Source**: Genotypes G1 to G24 -developed/maintained at ICAR-IIRR and the detailed QTL information is published in Mamidi et al., 2025 and G25 to G30- developed/maintained IRR, PJTAU.
