# Supplementary material for: A multi-model genotype × environment interaction analysis discerning phenotypic plasticity of the strong culm trait in rice
Source: Front Plant Sci. 2026 May 13;17:1727579. doi: 10.3389/fpls.2026.1727579 (PMC13212538; doi:10.3389/fpls.2026.1727579)
Supplement: Supplementary file 3 [file Table2.docx]

**Supplementary Table S2. Pooled mean of genotypes across multi-environmental conditions for all traits studied.**

| **Genotypes** | **PH** | **IL** | **CL** | **ODMa** | **ODMi** | **IDMa** | **IDMi** | **TN** | **PR** | **CD** | **CT** | **SM** | **BS** | **M** | **BR** | **CLSW** | **DFF** | **PN** | **PL** | **GN** | **PW** | **TW** | **GY** |
| --- | --- | --- | --- | --- | --- | --- | --- | --- | --- | --- | --- | --- | --- | --- | --- | --- | --- | --- | --- | --- | --- | --- | --- |
| **G1** | 118 | 12.6 | 100 | 8.39 | 6.55 | 5.55 | 4.57 | 10 | 18.70 | 7.78 | 2.56 | 29.48 | 70.73 | 1691 | 1075 | 12.99 | 130 | 9 | 26.2 | 284 | 4.69 | 20.79 | 563 |
| **G2** | 126 | 16.3 | 104 | 7.74 | 6.43 | 4.80 | 4.11 | 11 | 22.34 | 7.39 | 2.82 | 32.25 | 49.03 | 1266 | 1026 | 14.15 | 122 | 10 | 28.2 | 264 | 5.22 | 20.81 | 607 |
| **G3** | 105 | 12.8 | 84 | 7.19 | 6.16 | 5.11 | 4.07 | 10 | 25.33 | 6.95 | 2.22 | 23.40 | 74.41 | 1386 | 908 | 10.70 | 123 | 8 | 24.5 | 239 | 4.77 | 19.36 | 569 |
| **G4** | 122 | 13.5 | 99 | 7.01 | 5.60 | 5.12 | 3.88 | 11 | 26.49 | 6.56 | 1.91 | 18.45 | 63.10 | 941 | 910 | 13.96 | 129 | 10 | 24.4 | 241 | 3.54 | 27.86 | 572 |
| **G5** | 96 | 11.6 | 76 | 5.90 | 5.07 | 4.43 | 3.43 | 10 | 25.93 | 5.79 | 1.68 | 13.42 | 68.83 | 804 | 656 | 10.45 | 128 | 9 | 25.7 | 238 | 5.56 | 21.45 | 604 |
| **G6** | 121 | 13.8 | 98 | 6.37 | 4.89 | 4.58 | 3.76 | 10 | 26.10 | 5.85 | 1.51 | 11.63 | 63.27 | 618 | 873 | 12.98 | 125 | 9 | 20.8 | 242 | 3.66 | 17.01 | 585 |
| **G7** | 96 | 7.6 | 74 | 6.26 | 4.99 | 4.73 | 3.44 | 10 | 13.68 | 5.96 | 1.67 | 14.20 | 27.23 | 287 | 741 | 10.94 | 130 | 10 | 21.4 | 211 | 3.35 | 19.78 | 496 |
| **G8** | 101 | 6.5 | 74 | 7.84 | 6.25 | 5.70 | 4.08 | 9 | 24.34 | 7.38 | 2.27 | 26.17 | 68.25 | 1561 | 1065 | 11.08 | 129 | 9 | 23.2 | 219 | 4.55 | 22.66 | 564 |
| **G9** | 94 | 10.7 | 73 | 7.65 | 5.93 | 6.04 | 4.09 | 16 | 20.40 | 7.20 | 1.92 | 21.77 | 33.44 | 648 | 640 | 13.83 | 124 | 14 | 22.0 | 254 | 3.15 | 16.74 | 529 |
| **G10** | 108 | 11.6 | 77 | 5.71 | 4.63 | 4.28 | 3.49 | 11 | 20.07 | 5.91 | 1.54 | 13.64 | 46.35 | 588 | 798 | 8.70 | 111 | 9 | 23.5 | 214 | 3.94 | 20.64 | 437 |
| **G11** | 104 | 14.5 | 82 | 7.22 | 6.01 | 4.89 | 4.01 | 8 | 25.15 | 6.81 | 2.18 | 21.42 | 81.93 | 1506 | 1107 | 13.38 | 128 | 8 | 23.3 | 243 | 4.30 | 25.39 | 524 |
| **G12** | 123 | 15.2 | 98 | 6.94 | 5.25 | 4.31 | 3.59 | 10 | 27.77 | 6.57 | 2.47 | 20.00 | 64.88 | 1183 | 884 | 12.20 | 119 | 9 | 22.8 | 214 | 3.83 | 18.47 | 510 |
| **G13** | 116 | 12.3 | 90 | 7.32 | 5.99 | 5.39 | 4.15 | 12 | 21.91 | 6.87 | 1.89 | 20.65 | 60.11 | 1112 | 975 | 13.59 | 131 | 10 | 23.5 | 196 | 4.11 | 26.84 | 583 |
| **G14** | 101 | 7.9 | 78 | 7.89 | 6.46 | 5.10 | 4.08 | 12 | 32.57 | 7.51 | 2.65 | 24.43 | 73.83 | 1736 | 1130 | 12.02 | 123 | 10 | 27.8 | 226 | 4.02 | 20.97 | 582 |
| **G15** | 114 | 5.5 | 88 | 6.97 | 5.89 | 4.94 | 3.84 | 10 | 27.46 | 6.63 | 2.05 | 21.38 | 59.58 | 1170 | 1065 | 10.72 | 129 | 10 | 25.3 | 238 | 3.99 | 17.34 | 564 |
| **G16** | 90 | 11.0 | 73 | 5.42 | 4.78 | 4.29 | 3.85 | 12 | 29.46 | 5.25 | 0.99 | 7.30 | 53.52 | 361 | 760 | 8.61 | 131 | 12 | 19.9 | 232 | 5.04 | 21.47 | 635 |
| **G17** | 106 | 11.0 | 83 | 5.72 | 4.94 | 4.42 | 3.60 | 14 | 25.13 | 5.52 | 1.31 | 13.26 | 36.21 | 490 | 778 | 12.99 | 122 | 13 | 21.8 | 193 | 4.19 | 19.32 | 572 |
| **G18** | 108 | 11.0 | 80 | 6.38 | 5.51 | 5.29 | 3.90 | 12 | 23.61 | 6.12 | 1.39 | 15.94 | 47.70 | 709 | 995 | 10.40 | 111 | 11 | 22.0 | 231 | 4.47 | 17.27 | 517 |
| **G19** | 99 | 10.9 | 71 | 6.75 | 5.32 | 4.69 | 3.44 | 17 | 16.56 | 6.39 | 2.20 | 20.17 | 23.59 | 436 | 879 | 9.91 | 107 | 16 | 22.2 | 239 | 3.42 | 14.86 | 522 |
| **G20** | 113 | 16.3 | 85 | 6.43 | 5.56 | 5.01 | 4.29 | 11 | 22.44 | 6.27 | 1.82 | 17.38 | 60.10 | 946 | 1085 | 12.92 | 125 | 10 | 24.3 | 185 | 3.41 | 25.85 | 586 |
| **G21** | 91 | 10.0 | 57 | 3.05 | 2.48 | 2.59 | 1.80 | 21 | 11.53 | 2.68 | 0.59 | 1.36 | 12.84 | 17 | 169 | 4.91 | 148 | 19 | 22.6 | 236 | 3.93 | 13.61 | 462 |
| **G22** | 114 | 12.4 | 79 | 6.04 | 5.22 | 4.77 | 3.27 | 11 | 24.32 | 5.38 | 1.66 | 15.01 | 52.68 | 843 | 1082 | 9.89 | 137 | 10 | 26.8 | 230 | 4.97 | 19.31 | 532 |
| **G23** | 119 | 10.9 | 68 | 7.61 | 6.24 | 6.32 | 4.06 | 17 | 22.73 | 6.79 | 1.97 | 25.78 | 32.27 | 922 | 1094 | 15.09 | 134 | 14 | 27.8 | 247 | 6.57 | 22.69 | 569 |
| **G24** | 100 | 7.3 | 74 | 6.95 | 5.87 | 5.19 | 4.05 | 9 | 20.49 | 6.21 | 1.92 | 19.61 | 56.17 | 1200 | 754 | 11.07 | 117 | 9 | 23.1 | 270 | 3.00 | 18.85 | 519 |
| **G25** | 103 | 10.8 | 65 | 6.22 | 5.55 | 4.07 | 3.23 | 9 | 16.19 | 5.66 | 1.35 | 15.95 | 50.66 | 914 | 562 | 5.48 | 115 | 9 | 22.7 | 184 | 2.58 | 23.37 | 456 |
| **G26** | 94 | 10.0 | 54 | 5.89 | 5.59 | 5.08 | 3.72 | 15 | 13.60 | 5.79 | 1.71 | 13.59 | 22.46 | 301 | 499 | 9.80 | 113 | 12 | 22.3 | 254 | 5.25 | 24.48 | 524 |
| **G27** | 96 | 11.7 | 59 | 5.02 | 4.59 | 4.50 | 3.49 | 13 | 12.74 | 5.38 | 1.70 | 10.85 | 23.05 | 341 | 513 | 5.27 | 112 | 11 | 24.9 | 242 | 3.72 | 14.80 | 473 |
| **G28** | 104 | 9.1 | 54 | 6.57 | 5.45 | 4.76 | 3.74 | 15 | 17.01 | 5.67 | 1.44 | 11.73 | 29.03 | 362 | 949 | 8.43 | 111 | 11 | 24.3 | 247 | 4.62 | 24.40 | 566 |
| **G29** | 97 | 10.6 | 48 | 5.46 | 4.99 | 4.96 | 3.75 | 14 | 12.17 | 5.10 | 1.33 | 10.20 | 22.25 | 235 | 627 | 5.56 | 103 | 11 | 20.8 | 223 | 4.09 | 22.86 | 474 |
| **G30** | 92 | 10.2 | 45 | 4.99 | 4.22 | 4.03 | 3.18 | 15 | 11.61 | 4.62 | 1.11 | 7.96 | 19.08 | 161 | 713 | 5.10 | 100 | 11 | 20.3 | 243 | 3.02 | 13.93 | 431 |

PH, Plant height(cm); IL, Internode length(cm); CL, culm length(cm); ODMa, Outer diameter of major axis(mm); ODMi, Outer diameter of minor axis(mm); IDMa, Inner diameter of major axis(mm); IDMi, Inner diameter of minor axis(mm); TN, Tiller number; PR, Pushing resistance; CD, Culm diameter(mm); CT, Culm thickness(mm); SM, Section Modulus(mm^3^); BS, Bending stress(g mm^-2^); M, Bending moment at breaking(g cm); BR, Breaking resistance(g); CLSW, Culm with leaf sheath weight(g); DFF, Days to fifty percent flowering; PN, Panicle number; PL, Panicle length(cm); GN, Grain number; PW, Panicle weight(g); TW, Test weight(g); GY, grain yield(g).

**Supplementary Table S3. Annichiarico environmental index**

| **ENV** | **BR Mean** | **Winning genotype** | **Annichiarico environmental index** | **class** |
| --- | --- | --- | --- | --- |
| E1 | 867.92 | G22 | 20.8 | favorable |
| E2 | 843.64 | G22 | -3.4 | unfavorable |
| E3 | 901.29 | G15 | 54.2 | favorable |
| E4 | 775.33 | G18 | -71.7 | unfavorable |
| **ENV** | **GY Mean** | **Winning genotype** | **Annichiarico environmental index** | **class** |
| E1 | 547.11 | G2 | 15.4 | favorable |
| E2 | 520.62 | G28 | -7.0 | unfavorable |
| E3 | 530.22 | G16 | 2.5 | favorable |
| E4 | 513.67 | G16 | -10.9 | unfavorable |

ENV-environment; BR-breaking resistance; GY-grain yield
