# Supplementary material for: A multi-model genotype × environment interaction analysis discerning phenotypic plasticity of the strong culm trait in rice
Source: Front Plant Sci. 2026 May 13;17:1727579. doi: 10.3389/fpls.2026.1727579 (PMC13212538; doi:10.3389/fpls.2026.1727579)
Supplement: Supplementary file 4 [file Table3.docx]

**Supplementary Table S4. Scores for the 30 genotypes (G1-G30) and for the ideotype (ID) estimated in the first six factors.**

| **GEN** | **FA1** | **FA2** | **FA3** | **FA4** | **FA5** | **FA6** |
| --- | --- | --- | --- | --- | --- | --- |
| G1 | 9.01 | 6.50 | 2.63 | -2.20 | 0.46 | 1.21 |
| G2 | 7.08 | 5.98 | 3.53 | -1.40 | 0.88 | 0.89 |
| G3 | 6.97 | 4.16 | 2.98 | -3.05 | 0.34 | 1.15 |
| G4 | 6.91 | 5.78 | 2.38 | -0.56 | 0.16 | 1.51 |
| G5 | 6.46 | 5.23 | 3.63 | -2.14 | -1.40 | 2.02 |
| G6 | 5.50 | 4.60 | 1.76 | -2.21 | -0.21 | 2.31 |
| G7 | 7.09 | 4.70 | 2.98 | -1.12 | -1.69 | -0.18 |
| G8 | 7.96 | 4.65 | 2.74 | -1.43 | -3.19 | 1.64 |
| G9 | 6.86 | 6.06 | 1.01 | -0.73 | -2.24 | 0.82 |
| G10 | 5.85 | 4.06 | 2.64 | -1.51 | -1.54 | 0.68 |
| G11 | 6.95 | 3.92 | 3.29 | -1.44 | -1.53 | 1.93 |
| G12 | 6.71 | 5.80 | 1.59 | -1.95 | -0.45 | 2.09 |
| G13 | 7.79 | 4.32 | 3.27 | -0.62 | -1.60 | 1.09 |
| G14 | 7.42 | 4.96 | 2.74 | -1.46 | -2.84 | 2.33 |
| G15 | 7.57 | 6.47 | 1.78 | -1.17 | -2.82 | 2.82 |
| G16 | 4.86 | 5.29 | 5.03 | -1.72 | -2.28 | 2.17 |
| G17 | 6.14 | 6.58 | 2.82 | -0.42 | -0.84 | 0.99 |
| G18 | 5.85 | 5.89 | 2.87 | -2.17 | -1.22 | 1.83 |
| G19 | 7.39 | 5.85 | 1.72 | -2.67 | -2.09 | 0.37 |
| G20 | 6.42 | 3.03 | 3.17 | 0.59 | -1.08 | 0.15 |
| G21 | 4.27 | 7.16 | 2.20 | 0.43 | -1.41 | 0.50 |
| G22 | 6.54 | 5.71 | 2.19 | -1.09 | -1.36 | 1.55 |
| G23 | 8.00 | 5.59 | 3.94 | -1.19 | -1.80 | 0.52 |
| G24 | 7.50 | 5.15 | 1.74 | -1.86 | -2.05 | 1.19 |
| G25 | 6.17 | 3.43 | 0.30 | -1.38 | -0.85 | -0.38 |
| G26 | 7.30 | 6.83 | 4.22 | -1.53 | -1.58 | -1.52 |
| G27 | 7.05 | 5.68 | 1.76 | -1.81 | -2.35 | -0.64 |
| G28 | 5.81 | 5.27 | 3.80 | -2.93 | -2.08 | 0.38 |
| G29 | 6.44 | 5.03 | 2.97 | -3.53 | -1.50 | -0.12 |
| G30 | 5.27 | 5.88 | 1.75 | -3.72 | -2.03 | 0.55 |

G- genotype; FA factor retained; Bold values represent the selected genotypes.

**Supplementary Table S5. Selection gain for the mean of 23 strong culm and yield related traits**.

| **Trait** | **Factor** | **Xo** | **Xs** | **SD** | **SDperc** | **h2** | **SG** | **SGperc** |
| --- | --- | --- | --- | --- | --- | --- | --- | --- |
| **PH** | FA 2 | 105.76 | 115.13 | 9.37 | 8.86 | 0.88 | 8.25 | 7.80 |
| **IL** | FA 2 | 11.18 | 9.76 | -1.42 | -12.72 | 0.85 | -1.21 | -10.82 |
| **CL** | FA 2 | 76.24 | 82.14 | 5.90 | 7.74 | 0.88 | 5.19 | 6.81 |
| **ODMa** | FA 4 | 6.50 | 7.21 | 0.72 | 11.04 | 0.93 | 0.66 | 10.22 |
| **ODMi** | FA 3 | 5.41 | 6.07 | 0.65 | 12.09 | 0.94 | 0.61 | 11.31 |
| **IDMa** | FA 4 | 4.83 | 5.47 | 0.64 | 13.28 | 0.88 | 0.57 | 11.75 |
| **IDMi** | FA 4 | 3.73 | 4.05 | 0.31 | 8.37 | 0.90 | 0.28 | 7.53 |
| **TN** | FA 4 | 12.50 | 14.30 | 1.80 | 14.43 | 0.91 | 1.64 | 13.13 |
| **PR** | FA 1 | 21.26 | 23.62 | 2.36 | 11.11 | 0.90 | 2.13 | 10.00 |
| **CD** | FA 2 | 6.13 | 7.06 | 0.93 | 15.14 | 0.93 | 0.86 | 14.04 |
| **CT** | FA 2 | 1.83 | 2.17 | 0.34 | 18.71 | 0.93 | 0.32 | 17.39 |
| **SM** | FA 2 | 17.28 | 25.56 | 8.28 | 47.91 | 0.94 | 7.75 | 44.87 |
| **BS** | FA 1 | 48.22 | 50.26 | 2.04 | 4.23 | 0.93 | 1.91 | 3.96 |
| **M** | FA 2 | 824.56 | 1221.47 | 396.91 | 48.14 | 0.93 | 370.71 | 44.96 |
| **BR** | FA 2 | 847.05 | 992.56 | 145.51 | 17.18 | 0.91 | 131.96 | 15.58 |
| **CLSW** | FA 4 | 10.57 | 13.15 | 2.58 | 24.40 | 0.90 | 2.31 | 21.85 |
| **DFF** | FA 1 | 122.25 | 116.35 | -5.90 | -4.83 | 0.83 | -4.87 | -3.98 |
| **PN** | FA 4 | 8.33 | 10.09 | 1.76 | 21.13 | 0.90 | 1.59 | 19.11 |
| **PL** | FA 5 | 21.78 | 22.76 | 0.98 | 4.48 | 0.84 | 0.82 | 3.77 |
| **GN** | FA 2 | 218.31 | 262.45 | 44.14 | 20.22 | 0.87 | 38.21 | 17.50 |
| **PW** | FA 6 | 4.14 | 4.98 | 0.84 | 20.20 | 0.86 | 0.72 | 17.36 |
| **TW** | FA 6 | 20.31 | 23.33 | 3.02 | 14.86 | 0.87 | 2.62 | 12.92 |
| **GY** | FA 2 | 527.66 | 563.63 | 35.97 | 6.82 | 0.78 | 28.22 | 5.35 |

VAR, Variable; G- genotype; FA factor retained; (Xo), original average for WAASBY index; (Xs), mean for WAASBY index of the selected genotypes (G23, G1, G2, G15); SD, selection differential; SD perc, selection differential percentage; h2, heritability; SG, Selection gain; SG perc, Selection gain percentage; PH, Plant height(cm); IL, Internode length(cm); CL, culm length(cm); ODMa, Outer diameter of major axis(mm); ODMi, Outer diameter of minor axis(mm); IDMa, Inner diameter of major axis(mm); IDMi, Inner diameter of minor axis(mm); TN, Tiller number; PR, Pushing resistance; CD, Culm diameter(mm); CT, Culm thickness(mm); SM, Section Modulus(mm^3^); BS, Bending stress(g mm^-2^); M, Bending moment at breaking(g cm); BR, Breaking resistance(g); CLSW, Culm with leaf sheath weight(g); DFF, Days to fifty percent flowering; PN, Panicle number; PL, Panicle length(cm); GN, Grain number; PW, Panicle weight(g); TW, Test weight(g); GY, grain yield(g).
